# Theranostic Probes for Targeting Tumor Microenvironment: An Overview

**DOI:** 10.3390/ijms18051036

**Published:** 2017-05-11

**Authors:** Musafar Gani Sikkandhar, Anu Maashaa Nedumaran, Roopa Ravichandar, Satnam Singh, Induja Santhakumar, Zheng Cong Goh, Sachin Mishra, Govindaraju Archunan, Balázs Gulyás, Parasuraman Padmanabhan

**Affiliations:** 1 Lee Kong Chian School of Medicine, Nanyang Technological University, 59 Nanyang Drive, Singapore 636921, Singapore; musafargani030495@gmail.com (M.G.S.); mnmaashaa@gmail.com (A.M.N.); rupsravi@gmail.com (R.R.); saabsatnam01@gmail.com (S.S.); indup1893@gmail.com (I.S.); zgoh009@e.ntu.edu.sg (Z.C.G.); sachin.mishra@ntu.edu.sg (S.M.); 2 Centre for Pheromone Technology, Department of Animal Science, Bharathidasan University, Tiruchirappalli 620024, India; archunan@bdu.ac.in

**Keywords:** tumor microenvironment, nanoparticle, nanotheronostics, probe, imaging

## Abstract

Long gone is the time when tumors were thought to be insular masses of cells, residing independently at specific sites in an organ. Now, researchers gradually realize that tumors interact with the extracellular matrix (ECM), blood vessels, connective tissues, and immune cells in their environment, which is now known as the tumor microenvironment (TME). It has been found that the interactions between tumors and their surrounds promote tumor growth, invasion, and metastasis. The dynamics and diversity of TME cause the tumors to be heterogeneous and thus pose a challenge for cancer diagnosis, drug design, and therapy. As TME is significant in enhancing tumor progression, it is vital to identify the different components in the TME such as tumor vasculature, ECM, stromal cells, and the lymphatic system. This review explores how these significant factors in the TME, supply tumors with the required growth factors and signaling molecules to proliferate, invade, and metastasize. We also examine the development of TME-targeted nanotheranostics over the recent years for cancer therapy, diagnosis, and anticancer drug delivery systems. This review further discusses the limitations and future perspective of nanoparticle based theranostics when used in combination with current imaging modalities like Optical Imaging, Magnetic Resonance Imaging (MRI) and Nuclear Imaging (Positron Emission Tomography (PET) and Single Photon Emission Computer Tomography (SPECT)).

## 1. Introduction

Cancer is no longer considered as just a defect in the cell cycle control as a result of factors such as external radiation exposure, hereditary gene, or combinations thereof. Recent advancements in determining the factors that influence cancer development have indicated that even the immediate environment of the cancer cells influences their proliferation [1]. The cancer microenvironment, also known as the tumor microenvironment (TME), is an environment at the cellular level and consists of surrounding cells such as immune cells, inflammatory cells, extracellular matrix, and physical factors such as pH, temperature, hypoxia, interstitial fluid pressure, and others [2]. TME became a topic of interest for many researchers upon the discovery of the effects of immediate environmental changes such as lowering of pH and increased temperature on the proliferation of cancer cells. The contribution of TME is still undergoing research and recent studies suggest that both biochemical and physical cues in the environment significantly affect the growth and development of cancer cells [3].

One of the predominantly preferred methods to study and understand TME is molecular or metabolic imaging. Recent improvements in the imaging field have greatly impacted on the way in which TME is deciphered and has also improved the efficiency in monitoring dynamic interactions between tumor cells and their surrounding factors [4]. The pathway and function of imaging probes injected into the patient, are monitored by image guided therapy (IGT) for theranostic purposes. Apart from the tracking pathway and function, it also detects the progress of drug accumulation in the cells or even externally activates the specific nanoprobe with external means such as ultrasound, temperature etc. [5]. Although it is almost a well-established technique, there is still room for improvement in computer assisted surgery (CAS) as its ability is still limited [6].

Another way of monitoring a probe is using nanotheranostic techniques whereby probes in nanoscale are injected into the body for diagnostics and therapeutics or sometimes for both [7]. These probes have specific drugs and targeting moieties (peptides, antibody) attached to them, contributing to the specificity of the probe [8,9]. This has proven to be an efficient method to target the TME factors. The futuristic vision of this technique is to improve the personalized medicine concept, which is to customize medical procedures to suit the patient’s requirements.

One of the most widely used tools in nanotheranostics is use of nanoparticles (NPs). They come in various types, shapes and sizes according to target site, pathway and application. Each target has a few specific requirements that needs to be satisfied by the particular NP, so that the theranostic drug delivery system is efficient. Three main aspects need to be considered before choosing a type of NP interaction with the cells. First is the knowledge of the target cells and the biomarkers present in that target. Route of administration of the probe, that is, whether an oral administration or an intra venous (IV) injection, is the next thing to be considered. Finally, we have to consider the in vivo stability of the NP for the chosen purpose. For example, we need to consider whether the IV administered nanoprobe will be stable and can resist attack from the immune cells in the blood circulation until it is absorbed by the targeted cells. By using these guidelines to choose nanoprobes, many nanomaterials like nanodots, quantum dots, nanotubes, and nanowells have been designed.

Shape of the nano-probe is also an important factor to be considered. Although spherical nanoparticles are predominantly used in cancer theranostics, several non-spherical NPs like filamentous or worm like micelles, ellipsoid, nanorod, nanodisk etc. do have a number of favorable features including high drug loading capacity, long circulation time, better cellular uptake, longer tumor inhibition time etc. [10]. Banerjee et al. studied different nanoprobes with different sizes (50 and 200 nm) to test the effective transport and absorption in the intestine [11]. They demonstrated that the rod-shaped particle has higher cellular uptake and a more effective transportation system compared to spherical shaped. Another shape-based nanoparticle study was conducted by Loverde et al. using the Taxolanticancer drug in the block copolymer nanoparticle and which developed worm and sphere shaped hydrophobic particle coated with PEG-PCL, PEG-PLA and PEG-PBD. They concluded that the worm shaped NPs showed stronger hydrophobicity with PEG-PBD [12].

NPs have been proven to be a useful means for both early detection and drug delivery for cancer cells. Matrix metalloproteinases (MMPs) are hydrolases which are responsible for tumor growth, invasion, and metastasis. They also play a role in formation of tumor blood vessels. However, these have emerged as probable targets for early cancer detection. For example, matrix metalloproteinase (MMP-2) responsive nanoprobe is a type of NP developed to target breast, colon, and prostate cancer cells. MMP-2 plays an important role in the early detection of cancer due to its over expression on cancer cells compared to healthy cells [13]. Amongst the discussed nanoprobes, the most studied NPs for MRI are iron oxide and gold NPs [14].

**Figure 1:**
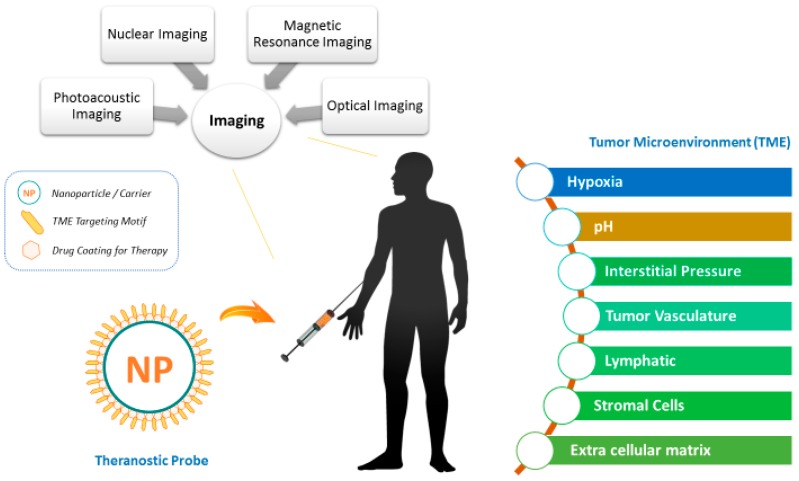
Illustration of theranostic probes incorporated for targeting the tumor microenvironment.

Most NPs either target the receptors on the cell, the intracellular target elements or the environmental factors specific to the cellular microenvironment [15]. Thus, the target system can be classified into physical factors such as hypoxia, pH, and interstitial fluid pressure and components of TME such as vasculature, lymphatic system, stromal cells etc. Low levels of oxygen have been proven to induce cancer characteristics in cells and similarly lower pH in tumor cells has been observed in comparison to healthy cells. Changes in interstitial pressure of the cells indicate an increase or decrease in cell permeability. Increased permeability in turn leads to influx or efflux of any foreign body in the cell, which might be a cancer carrier. It is very difficult to target the vasculature in the cancer environment due to increased permeability and loose membranes. Stromal cells are the cells surrounding the tumor which might influence the uncontrollable proliferation of the cancerous cells. Some of the stromal cells are cancerous fibroblasts and macrophages which indirectly induce cell division. Extracellular matrix (ECM) is another target in the TME which has been given consideration as the change in its mechanical property can prompt tumor formation also. Thus, this review aims to summarize TME-specific nano-theranostic probes that have been developed over the past few years designed for different imaging modalities, coupled with their usefulness and limitations [16]. Figure 1 shows the illustration of a theranostic probe incorporated for targeting the tumor microenvironment. Table 1 lists significant probes incorporated for therapy and imaging of the tumor microenvironment.

## 2. Tumor Microenvironment: Theranostic Probes for Physical Factors

The physical factors which greatly influence the proliferation of cancer cells are hypoxia, pH, and the interstitial pressure in the surrounding environment. These factors also serve as targets for the development of specific theranostic nanoprobes, which will be further discussed in this section.

### 2.1. Hypoxia

Hypoxia is a state of oxygen deprivation in the tissues. Thomlinson and Gray observed necrotic cells at a diffusion distance around 150 μm off the closest capillaries in human lung cancer cells [17]. They also realized the correlation between hypoxia and the failure of radiation therapy. It was only after many experiments that they discovered the failure was due to the diminished levels of free radicals in the hypoxic cells.

After four decades, it was discovered that the hypoxia-inducible factors (HIF) consisting of HIF-1 and HIF-2 activated gene transcription for the expression of the phenotypes were associated with cancer cells [18]. The hypoxic tumor cells can be caused by limited diffusion of oxygen (chronic) or vasculature collapse (acute) [19]. HIF binds to hypoxia response elements (HREs) to maintain the state of oxygen deficiency in cancer cells, so that they continue carrying out invasion, progression, metastasis, and hindrance to radiation therapy [20]. The early attempts to target hypoxia with radiation sensitizers and hyperbaric oxygen failed to be very precise, thereby requiring more precise optimization techniques [21]. Thus, the discoveries of therapeutic agents that inhibit HIF-1 are a prominent choice for cancer therapy. Examples of HIF inhibitors are HIF specific single interference RNA and antisense RNA that suppress the invasion and metastasis in pancreatic adenocarcinoma [22].

Several molecular imaging modalities targeting tumor hypoxia such as Positron Emission Tomography (PET), Single Photon Emission Computer Tomography (SPECT), Magnetic Resonance Imaging (MRI), and Optical Imaging have been developed. In the upcoming section, nanotheranostic probes targeting tumor hypoxia and their corresponding imaging modalities will be discussed.

**Figure 2:**
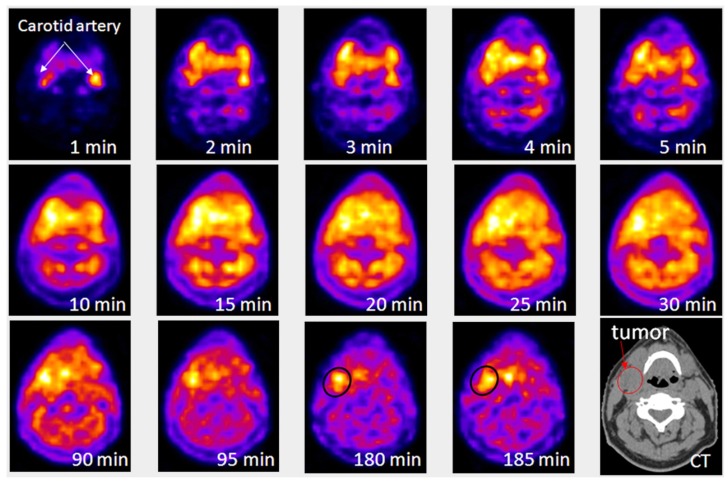
Results of a dynamic Positron Emission Tomography (PET) scan as a function of time after injection with ^18^F-fluoromisonidazole. A single reconstructed PET slice is displayed through the center of the head and neck tumor. First 5 frames were of 1-min duration, followed by 5 frames of 5-min duration. At 30 min after injection, the patient was removed from the scanner and then reimaged at 90 and 180 min after injection. Images were co-registered using a low-dose CT scan (depicted in the final image in the series). This series shows evolution of ^18^F-fluoromisonidazole distribution within the patient from initial blood pool to selective sequestration within the hypoxic tumor subvolume. This research was originally published in JNM. Carlin, Sean, and John L. Humm. PET of hypoxia: current and future perspectives. J. Nucl. Med. 2012, 53, (8), 1171–1174. © by the Society of Nuclear Medicine and Molecular Imaging, Inc. [26].

PET imaging of hypoxia for in vivo detection is based on the capability of the molecules to bind with the deoxygenated cells using the imaging probes namely, ^18^F-fluoromisonidazole (^18^F-FMISO), ^61^Cu-diacetyl-*bis*(*N*4-methylsemicarbazone) (^61^Cu-ATSM), ^18^F-flortanidazole (^18^F-HX4) etc. The FMISO PET determined the severity of tumors in a case study conducted on 22 patients with glioblastoma multiforme (GBM). It was found that the signal intensity of the image correlated with the severity of hypoxia in cancer cells [23,24]. In addition, ^61^Cu-ATSM PET elevated the contrast for intensity-modulated therapy in head and neck cancer, but this method required precise dosage of radionuclides attached to prodrug or theranostic agents [25]. Despite the advancement of all these probes, PET imaging to target hypoxia in tumors is still confined to the research domain. Figure 2 shows the results of a dynamic PET scan and evolution of ^18^F-fluoromisonidazole distribution within a patient from the initial blood pool to selective sequestration within the hypoxic tumor subvolume [26].

Magnetic Resonance Spectroscopy (MRS), has evolved with probes for determination of the level of deoxygenation using the relaxation of Hexamethyldisoloxane (HMDSO (^1^H MRS)). It has been used to measure the tension of oxygen in prostate cancer cells after intravenous injection [27,28]. Furthermore, perfluorocarbons (^19^F MRS) are used in combination with nitroimidazole to design a probe, namely 2-Nitro-α-[(2,2,2-trifluoroethoxy)methyl]-imidazole-1-ethanol (TF-MISO), that undergoes reduction when it binds to the endogenous molecules of hypoxic cells. This enables it to determine the severity of hypoxia in vivo [29]. Usually, the nitroimidazole-based probe involves the biomarker SR4554, which was experimented on 26 different patients with predominant gastrointestinal cancer [30]. To conduct surface coil acquisition of tumors using ^19^F MRS, the tumors must be less than 3 cm in diameter and less than 4 cm in depth. To improve the imaging modality, positive contrast enhancement is achieved using gadolinium-tetraazacyclododecane tetraacetic acid monoamide conjugate of 2-nitro-imidazole (GdDO3NI). Apart from measuring the oxygen deficiency in tumors, it was possible to image the area with low blood vessel perfusion in a xenograft rat model of prostate cancer [31].

Optical Imaging using fluorescent tags plays an important role in studying the hypoxia responsive elements (HRE) of carcinomatous cells [32]. It was tested on xenograft models to draw the relevance between vasculature and deprivation of oxygen. Additionally it was also observed in the experiment that hypoxia affects the transport of macromolecules, metabolism, and distribution of collagen-1 fiber [33]. However, the fluorescent imaging could not provide a more sensitive metabolic imaging in hypoxic tumors when compared to PET imaging.

Photoacoustic imaging is a hybrid technique which works on the principle of optical absorption and ultrasonic wave propagation. Electromagnetic radiation in the form of pulsed laser is irradiated on the sample which usually contains endogenous chromophores such as haemoglobin or deoxy haemoglobin or organic dyes and nanoparticles. This causes rapid heating and thermal expansion that leads to generation of acoustic waves which are detected by ultrasonic transducers. Since scattering of ultrasound is much less compared to light in human tissues, this leads to higher resolution images. Along with this, a higher contrast is obtained due to the use of the optical imaging technique. Hence, photoacoustic imaging generates high resolution and high contrast images [34]. An application of this technique can be seen by the various photoacoustic imaging probes sensitive to the oxygen level which have been developed for theranostic applications in targeting tumor microenvironment. It has evolved with targeted imaging and selective therapy of gastric cancer stem cells as an efficacious anticancer treatment. Shujing et al. designed gold nanostars (GNS)-based PEGylated multifunctional nanoprobes conjugated with CD44v6 monoclonal antibodies (CD44v6-GNS) as targeting ligands. Photoacoustic imaging revealed that this nanoprobe is capable of targeting the gastric cancer vascular system actively at 4-h post-injection, whereby the probes could also inhibit tumor growth remarkably upon NIR laser irradiation in gastric cancer bearing mice. Thus, the CD44v6-GNS nanoprobes have a great potential application for gastric cancer targeted imaging and photothermal therapy [35]. On the top of imaging modalities, the advancement in therapeutic genes namely, cytosine deaminase (CD) for HRE activation has been developed. The formation of the cytotoxic 5-fluorouracil (5 FU) from the interaction between nontoxic 5-fluorocytosine (5 FC) and CD can be imaged in vivo using ^19^F MRS. This method can be applied using NPs for the delivery of molecular reagents such as siRNA, cDNA, and mRNA in clinical trials [36].

### 2.2. pH Level

The balance between the extracellular and intracellular pH is achieved by a group of transporters such as Na^+^/H^+^ pumps and carbonic anhydrases (CAs) I-XIII [37]. They can stabilize the concentration levels of hydronium ions generated by cellular metabolism such as glycolysis. The acidic environment is due to the stringent blood perfusion and increased level of glycolytic activity in the tumor cells [38]. With the prior knowledge of abnormal pH in the TME, various theranostic probes are enumerated below. 

Firstly, MRS can monitor the chemical shift of the mild signals from inorganic phosphate to characterize the variation of the intracellular pH (pHi) using pH marker such as 3-aminopropylphosphonate. ^31^P MRS was used for characterization of extracellular pH (pHe). Unfortunately, the sensitivity of ^31^P MRS is low which limits the spatial resolution [39]. Thus, the spatial resolution for pHe could be increased in in vivo imaging by replacing the probes with ^1^H MRS namely (imidazol-1-yl)3-ethyoxycarbonylpropionic acid. Later, an improved spatial resolution with enhanced contrast for imaging the pHe was achieved using another ^1^H MRS probe namely, 2-(imidazol-1-yl) succinic acid with increased specificity and sensitivity [40].

Kato and Artemony developed a novel dual-tracer MRI-based theranostic technique to track the drugs released from nanocarriers using the SPION and gadolinium diethylenetriamine-penta-acetic acid bismethylamide (GdDTPA-BMA) [41]. The utilization of SPION implements a domineering negative enhancement over the positive enhancement due to GdDTPA-BMA, thus allowing pH-based theranostic probes to approach deep layered tissues [42].

Optical probes have been designed to image the acidification in tumors using a pH-activated near infrared fluorescent (NIRF) probe with an elevated sensitivity and quicker data acquisition. Optical imaging using NIRF probe enhances the sensitivity and spatial resolution because it reduces the absorbance and auto-fluorescence of endogenous molecules [43]. One of the probes called DilR 783-S can be activated in the acidic state by the breaking of hydrazine bonds, causing a fluorescence effect in the MDA-MB-435 xenograft model. 

However, the available probes have not been used in clinical trials so far. Furthermore, other pH probes like the chemical Exchange Saturation Transfer (CEST) has been used to image breast cancer in a xenograft model using contrast agents of CT such as iopromide [44]. It can determine the correlation between the amide protons of iopromide and the level of pH. The pH was observed to be in the narrow range of 6.3 to 7.2 irrespective of concentration. The pH level of the tissues could also be estimated through hyperpolarized H^13^CO_3_ with prior intravenous injection in in vivo detection [45]. The calculation of pH was done using the Henderson-Hasselbalch equation and it was applied in the lymphoma model of the preclinical trial.

Besides the use of imaging probes to track the fatality of cancer, there is an urgent need for the design of pH-sensitive carriers for a more specific and effective targeting. Polymeric micelles are well-known pH-sensitive hydrophobic carriers used to target tumors with great specificity because they contain ionizable groups [46]. The hydrophobic drugs can be encapsulated in micelles to function in the acidic state of TME. The typical doxorubicin-encapsulated pH responsive polymeric micelles have been observed to release the maximum of 70% of the target molecule or drug at a pH level of 6.4, while it stops at a pH of 7.4 [47]. The ability of these polymeric micelles to detect the antitumor region has been experimented in vivo.

Adding to the list, photoacoustic (PA) imaging is one of the emerging imaging modalities utilized in the field of theranostics for targeting tumor microenvironment. In recent times, Ying et al. have developed peptide functionalized gold nanostars (GNS) for photoacoustic tomography. This desirable targeting efficiency has improved the effect of Photothermal Theraphy (PTT) without damaging the adjacent tissues [48]. In the case of phantom PA imaging, the signals acquired from the GNS-pHLIP (Gold Nanostars-pH Lipid) were imaged using the photoacoustic 3-D tomographic system (Endra Nexus 128, Ann Arbor, MI, USA) at an absorption wavelength of 808 nm. Also, in a recent study, various photoacoustic probes were studied by B Shi et al. One such probe was used for real time tracking of endogenous H_2_S in HCT116 mice cell. This probe had a local concentration of Butylated, Octylated Diphenylamine (BODPA) within the NPs which is activated at pH 7.4 [49]. Although PA imaging provides extensive theranostic applications for targeting the tumor microenvironment, only limited probes have been developed so far.

### 2.3. Interstitial Fluid Pressure (IFP)

Imbalance in the angiogenic factors like vascular endothelial growth factor (VEGF), matrix metalloproteinase (MMP) and angiopoietins leads to the production of abnormal vasculature. Sometimes, the hyperpermeability allows exchange of molecules in the tumor vasculature without any maintenance of gradient. This gives rise to an abnormality in IFP [50].

Non-invasive imaging methods like dynamic contrast enhanced magnetic resonance imaging using Gd-DTPA (gadolinium diethylene-triamine penta-acetic acid) are helpful to analyze IFP, and pre-clinical study using xenograft mouse models of many human cancers was performed. It was concluded that, the velocity with which fluid flows correlates well with the magnitude of IFP [51]. Liu et al. discussed an extended study of this principle to clinical non-invasive studies on human liver tumors for measurement of IFP as a function of three parameters i.e., velocity of tumor interstitial fluid, distance between tumor surface and exudates, and conductivity of the interstitium. The research also discussed a method to non-invasively measure IFP using these parameters [52]. 

Nanoprobes designed for targeted therapy towards this abnormal IFP would help to suppress the rapid angiogenic process. A pre-clinical study of a probe designed to target and inhibit IFP on a mouse B16 melanoma model was performed using sterically stabilized lysosomes filled with Imatinib, a drug to reduce IFP by inhibiting platelet-derived growth factor receptor (PDGF-R) β. These lipid vehicles were distributed in the tumor. A reduction of tumor size was observed under fluorescence imaging [53]. Development of theranostic probes targeting IFP is an ongoing research and hence not many probes have been reported. However, there are many therapeutic drugs such as Imatinib and Anakinra (Interleukin antagonist) that decrease the IFP and increase the drug uptake. On the other hand, the effect of these drugs has to be studied carefully and hence hybrid imaging like PET/CT takes a unique role. A research on water-perfusable tissue fraction (PTF) with help of ^15^O-water PET/CT helped to analyze the outcome of Imatinib on the IFP in colorectal cancer [54]. 

## 3. Tumor Microenvironment: Theranostic Probes for Physical Factors

TME is a key player in the development and progression of cancer. It can be targeted to suppress the severity of cancer. The need for nanoprobes has become important in current research because of the limitations of conventional therapies for cancer treatment. The following section will give an insight into the various therapeutic and imaging probes developed so far to target various components of TME.

### 3.1. Tumor Vasculature

When a tumor is developing, it needs to be supplied with new blood vessels. The vessel formation (angiogenesis) does not happen sequentially and hence it has increased permeability and leaky membranes. Because of this malformed vasculature, it becomes difficult to have drug delivery. Hence, these days nano-therapeutic applications have emerged to tackle the drug delivery inefficiency [50]. 

The nano-probe contains photofrin or photofrin and iron oxide NPs. Photofrin is a photodynamic agent which acts like a sensitizer that is taken up by the tumor and upon photo irradiation releases singlet oxygen [55]. These NPs are tagged with an F3 peptide which specifically targets the surface of MDA-435 cells. This can be visualized due to tagging of Alexa Fluor 594 to the NP. Pre-clinical studies in a rat glioma model using this probe showed better tumor localization and contrast enhancement in MRI for the targeted agent compared to non-peptide tagged NPs. Moreover treatment of rat glioma using Photo Dynamic Therapy (PDT) gave improved results for the peptide tagged NP. This study was performed using diffused MRI with 2D reconstruction [55]. A probable disadvantage is that photofrin remains in the skin for a long time, causing the patients’ skin to be photosensitive for weeks. The tissue penetration for this probe could be difficult due to the presence of photofrin. Advancements such as use of SPION with a siliceous coating and a tagged fluorophore can increase the specificity towards the tumor target. Moreover, the use of a nickel magnetic micromesh which can be magnetized using a strong external magnetic field can target the fluorescent magnetic nano-particles (FMN) which in turn enhances the cancer targeting [56]. With the help of magnetic NPs, the size and distance constraints for targeting the required tissue are reduced.

Schmider et al. later developed a probe to target neo-vasculature expressing α_5_β_1_ integrin and these vessels were visualized with 3D spatial resolution of the MR signals. They did another experiment with a theranostic nanoprobe targeted towards α_5_β_1_ (α_ν_β_3_) integrin along with a fumagillin drug. Results showed a significant reduction in tumor size compared to α_ν_β_3_ fumigillin which did not show much reduction [57]. The advantage of using 3D reconstructions of the MR signal is that, we can clearly depict the volume of tumor reduction to accurately identify the therapeutic effect of the probe. This research study was carried out at a pre-clinical level using Α5β1 integrin in the MDA-MD 435 xenograft mouse model. 

Increasing the specificity of a nanoprobe by adding a peptide can be seen from the research conducted by Grange et al. where neural cell adhesion molecule (NCAM) expressed on tumor endothelial cells or Kaposi Cells can be particularly targeted with a specific NCAM peptide conjugated with a nanoprobe. A comparison was made between a PEG liposome with no specific NCAM target and C3d liposome which had a specific peptide moiety. They concluded that C3d liposome was more efficient in delivering the doxorubicin drug into the tumor cell which resulted in a reduction in tumor size and also with less toxicity of the drug and this was observed on SCID male mice at a pre-clinical level. On the other hand, the PEG liposome was accumulated extracellularly. The doxorubicin drug release was observed through MRI by tagging the liposome with a gadolinium—1,4,7,10-tetraazacyclododecane-1,4,7,10-tetraacetic acid (DOTA)—monoamide or Gd DOTAMA (C18)_2_ in short [58]. Gd DOTAMA, an important contrast agent, is capable of determining specific signatures of the TME. 

Similarly, another study with Gd DOTAMA as a contrast agent in targeting tumor vasculature where a glucocorticoid drug prednisolone phosphate (PLP) was incorporated inside long circulating stealth liposomes (LCL) and this combination of LCL-PLP could permeate through the tumor vasculature easily compared to normal endothelial cells to achieve a targeted therapeutic effect. Two kinds of drug release study were conducted: one with only LCL-PLP and other with an MRI imaging agent Gd-PLP-LCL. The latter helped to understand the distribution of the drug delivered in vivo on B16.F10 melanoma cells injected into Male C57BI6 mice and concluded that this had a good accuracy in analyzing the therapeutic response at a pre-clinical level [59].

**Figure 3:**
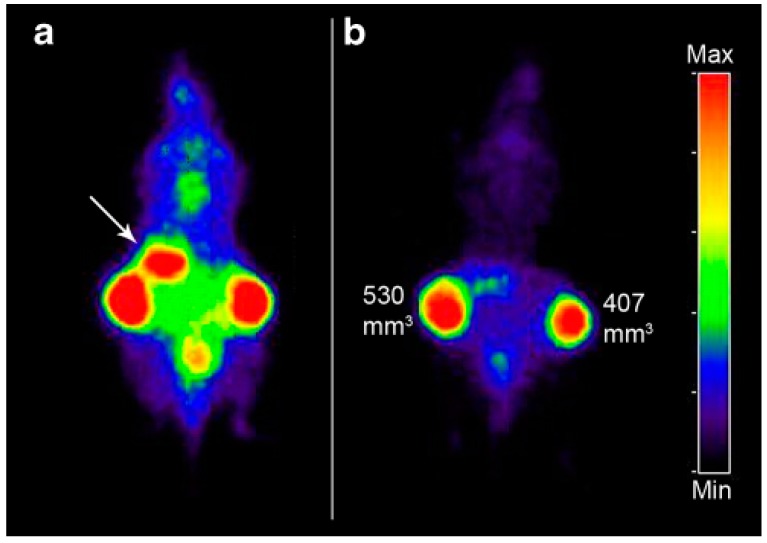
PET images of FaDu xenograft-bearing nude mouse injected with ^124^I-L19-SIP (3.7 MBq, 25 μg). Coronal images were acquired at 24 (**a**) and 48 h (**b**) after injection. Image planes were chosen where both tumors were visible. Uptake of ^124^I in the stomach (arrow) and to some extent in bladder (urine) is visible at 24 h p.i., but has disappeared at 48 h p.i. This research was originally published in European Journal of Nuclear Medicine and Molecular Imaging. Bernard M. Tijink. ^124^I-L19-SIP for immune PET imaging of tumour vasculature and guidance of ^131^I-L19-SIP radioimmunotherapy © 2016 Springer International Publishing AG [62].

VEGF regulates tumor angiogenesis and is over-expressed in a tumor tissue. The usage of many other imaging modalities such as PET and SPECT in targeting the VEGF is evident from the following examples which were performed at a pre-clinical stage. ^124^I-VG67e is a monoclonal antibody that binds to VEGF-A that can be imaged through PET and this was done on HT1080-26.6-bearing mice at a pre-clinical stage [60]. To target the VEGF receptor, probes such as ^64^Cu-DOTA-VEGF_121_ are performed on mice bearing U87MG human glioblastomas where DOTA acts as a chelator [61]. 124-L19-SIP monoclonal antibody was targeted against extra domain-B of fibronectin on xenograft nude mice [62] (as shown in Figure 3). SPECT imaging modality is also useful for detecting angiogenesis markers with the help of probes like 99mTc-scVEGF targeted towards the VEGF receptor of Male Swiss–Webster mice [63]. VEGF121-Avi-streptavdidn IRDye800scVEGF/Cy is a probe which uses optical imaging or NIRF for detecting VEGF receptor performed on Mice bearing VEGFR-2 expressing 67NR murine breast tumors [64].

A study by Shi et al. explained the use of reduced graphene oxide conjugated with the antibody of CD105 which is predominantly expressed on growing tumor cells. This led to formation of RGO conjugate ^64^Cu-NOTA-RGO-TRC105 showing stability in vitro and in vivo. PET imaging studies assessed the pharmacokinetics and selectivity of the conjugate towards CD105 on 4T1 murine breast tumors and this was also validated with histologic studies [65]. The same team further investigated on to target VEGFR using NOTA-GO-VEGF121 which incorporated nano-graphene oxide sheets and performed in vivo mouse PET imaging studies which proved the specificity of this conjugate to VEGFR. This has been predicted to play an important role in cancer theranostics [66]. 

Dual Modality imaging using both PET/NIRF was carried out using ^64^Cu-MSN-800CW-TRC105(Fab) which used multifunctional mesoporous silica nanoparticles and 800CW which acts as the NIRF dye. This experiment was performed on the 4T1 murine breast tumor model and resulted in a 2-fold enhancement of tumor accumulation compared to targeting without TRC105(Fab) [67]. In vivo ImmunoPET imaging study of tumor bearing mice with ^124^I MORAb-004 has shown to specifically target hTEM1 which is a tumor vascular marker and this was expressed by engineering immortalized murine endothelial cells (MS1) to express luciferase, enabling optical imaging in nude mice [68].

A multifunctional theranostic approach involving photoacoustic imaging was performed to image tumor vasculature associated with mouse bearing the 4T1 breast tumor model using (PEG) coated copper(II) sulfide NP. These NPs act as a contrast agent and also help to destroy the tumor by photothermolysis [69].

Clinical PET Imaging studies have also been done for example by using ^89^Zr bevacizumab, a tracer which helps to target VEGF-A in breast cancer patients. This study showed that the standardized uptake value of the tracer was higher (1.85 ± 1.22) in the tumor compared to normal tissues (0.59 ± 0.37) [70]. ^123^I-L19(scFv)_2_ targeted towards fibronectin of five male patients with head and neck cancer could be imaged by SPECT/CT modality [71].

### 3.2. Lymphatic System

The lymphatic system plays an important role in both the immune system and the spreading of cancerous cells from the primary site to the secondary site in different organs, i.e., cancer metastasis. In many diseases including cancer, there is an excess growth of lymphatic vessels called lymphangiogenesis [72]. Lymphatic endothelial cells express specific antigens such as podoplanin, lymphatic vessel endothelial hyaluronan receptor 1 (LYVE-1) and VEGFR3. LYVE-1 is the most extensively used lymphatic endothelial cell marker and probes for targeting lymphatic cells have been developed for PET and optical imaging [73,74,75]. The presence of podoplanin on lymphatic endothelial cells promotes their migration, adhesion, and lymphatic formation. Yang et al. developed a nanoprobe to target podoplanin in lymphangiogenesis. They developed polyethylene glycol (PEG)-GoldMag NPs which were conjugated with anti-podoplanin antibody (PodAb) which was later evaluated for the occurrence of tumor lymphangiogenesis in vivo using MRI in breast cancer model [76].

**Figure 4:**
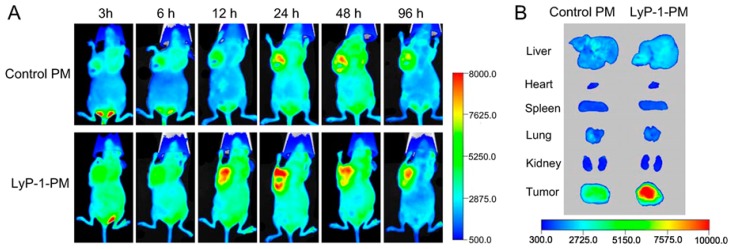
The targeted delivery of LyP-1-PM to highly metastatic breast tumor in vivo. (**A**) In vivo near-infrared fluorescent images of mice after intravenous administration of DiD-loaded LyP-1-PM or PM at different time points and (**B**) ex vivo image of tumors and organsafter the tumor-bearing mice above were sacrificed at 96 h. Reprinted with permission from [80]. Copyright 2009 American Chemical Society.

Lymphatics-homing peptide-1 (LyP-1) is a 9-amino-acid cyclic peptide that binds to its receptor (p32/gC1qR) in some type of tumor cells especially, in highly malignant breast tumor cells MDA-MB-231 and MDA-MB-435S [77]. Luo et al. synthesized a NP-specific to tumor lymphatics conjugated with LyP-1 [78]. They used copolymers of maleimide–PEG–poly(lactic-co-glycolic) acid (PLGA) which were conjugated with fluorescein isothiocyanate (FITC) labelled LyP-1. They found an eight times higher uptake of LyP-1-NPs in vivo than without LyP-1 NPs, used for comparing efficacy. The detection of FITC positive cells was done with fluorescence microscopy. In 2011, Zhang et al. also, labelled LyP-1 peptide with Cy5.5 (a near-infrared fluorophore) for visualizing under optical imaging [79]. Later in 2012, Wang et al. prepared another NP with LyP-1 conjugated PEG-PCL micelles (LyP-1-PM) and Artemisinin (ART) (a natural anti-cancer and anti-lymphangiogenesis drug). Micelles with artemisinin drug (LyP-1-PM-ART) were observed to have higher antitumor efficacy. LyP-1-PM-ART was found to specifically target lymphatic tumor tissue as compared to PM-ART, which remained in the circulation. They showed near-infrared fluorescent imaging in vivo as well as ex vivo and observed that 10-dioctadecyl-3,3,30,30-tetramethylindodicarbocyanine-4-chlorobenzene-sulfonate salt (DiD)-loaded LyP-1-PM (LyP-1-PM-DiD) showed preferential accumulation in the tumor than PM-DiD (as shown in Figure 4). These findings suggest that the LyP-1 modified fluorescence probe has theranostic application to highly metastatic breast tumor and tumor lymphatics [80].

### 3.3. Extracellular Matrix

Changes in the rigidity of the ECM can be a trigger for tumor development. Therefore, when nanocarriers are targeted to this lineage of TME to stop these changes associated with ECM, the growth of the tumor can be suppressed. 

Hyaluronan (HA) metabolism is a predominant feature for breast cancer progression. During tumor progression, the amount of hyaluronidases is at increased levels. Peer and Margalit discussed HA tagged to gold NPs along with a fluorophore which helps to detect the cancerous tissue due to increased levels of hyaluronidases [81].

A nanotherapeutic probe targeting ECM was designed by attaching an antibody of lysyl oxidase to poly(d,l-lactide-co-glycolide)-block-PEG copolymer. This nanoprobe system was successfully implemented in vitro and in vivo and resulted in significant tumor suppression. The research team conducted a fluorescence spectroscopic analysis using Coumarin-6 dye that helped to analyze the nanoprobe accumulation inside the tumor [82]. 

Proteases that are released by stromal cells help to give property of shape to ECM in tumor cells. These proteases are released excessively and cause the degradation of ECM. Probes targeting the activity of protease help to image tumor progression using Fluorescence Resonance Energy Transfer (FRET) and NIR imaging [83].

Matrix metalloproteinase (MMP-2) is usually over expressed in certain tumors. A probe that follows FRET principle was designed by attaching a low molecular weight heparin associated with quantum dot and this moiety was attached to the low molecular weight protamine which contains the MMP-2 substrate cleavable by MMP-2. This substrate acts as a spacer and also contains the fluorophore. MMP-2 overexpressed in the tumor tissue cleaves this spacer leaving the fluorophore free. To make this probe permeable through the blood brain barrier (BBB), a transferrin receptor T7 peptide was used for targeting the tumor correctly [84].

A recent advancement in this field was the use of an external alternate magnetic field which makes the liposomes covered by heat sensitive magnetic NPs undergo hysteresis and this localized increase in temperature triggers the release of protease substrates embedded inside the liposome. MMPs excessively present on tumor tissues degrade the substrate and these short peptides are detected by diagnostic tests like enzyme-linked immunosorbent assay (ELISA) [85].

Collagen undergoes many changes in the TME. These structural proteins become denatured due to action by MMPs and several other proteases. Collagen mimetic peptides (CMP) are photo-triggered and have a tendency to form a triple helix with the denatured collagen. The CMPs were tagged with Near Infrared Fluorophore (NIRF) (IR-Ahx-(GPO)_9_) and fluorescence revealed the accumulation of the triple helical structure in the tumor tissue [86].

An extracellular matrix protein named periostin has functions of cell adhesion and motility and is an important component of TME. This protein was imaged using PET/CT modality. A PET tracer ^64^Cu-DOTA-antiperiostin-F(ab′)2 was developed by Heidari et al. which showed better binding affinity towards periostin expressed in esophageal squamous cell carcinoma mouse models in comparison with ^18^F FDG [87].

### 3.4. Stromal Cells

Tumor-associated stromal cells (TASCs) such as cancer-associated fibroblasts (CAFs) and tumor-associated macrophages (TAMs) have been found to play a key role in promoting cancer progression and metastasis [88,89,90]. CAFs are widely seen in breast and pancreatic cancer [91,92]. The overexpression of fibroblast activation protein-α (FAP) (membrane bound serine protease), on the CAFs, makes them different from the normal stromal cells [93]. The non-specific binding of FAP, due to sharing peptide substrate with other postpropyl peptidase, is the limitation for the fabrication of FAP targeted imaging probe for in vivo studies. To counter this limitation, Granot et al. labelled the CAFs in vitro with the contrast agent biotin-bovine serum albumin-gadolinium diethylenetriaminepentaacetic acid, Feridex, or 1,1′-dioctadecyl-3,3,3-tetramethylindotricarbocyanine iodide. Henceforward, they could be easily traced by MRI or NIRF in vivo imaging [94,95]. FAP has a unique post-prolyl endopeptidase activity to cleave the dipeptide at the NH_2-_terminal [96,97]. Hence, FAP-α is known to give shape to the microenvironment which promotes growth as well as invasion of the tumor by degrading ECM [94,98,99]. Ji et al. designed the ferritin-based FAP-α responsive fluorescence probe for imaging of CAF-positive cancers. Since FAP-α is excessively present in tumor tissue, the ferritin-based fluorescence-tagged peptide on the probe will become cleaved by the FAP-α to release the fluorophore and be detected. In vivo imaging also was performed by Ji and colleagues in mice after injecting these nanoprobes intravenously [100]. Ji et al. also designed a novel nanocarrier made up of a cleavable amphiphilic peptide (CAP), encapsulated with hydrophobic drug doxorubicin (Dox) to target CAFs in the TME. This CAP-Dox-NPs showed excellent results in terms of both specificity and antitumor activity in vivo when examined by in vivo fluorescence imaging [101].

Furthermore, cysteine cathepsins especially cathepsin B (CtsB) are excessively expressed in TME. Normally, CtsB interacts with lysosomes to perform functions such as autophagy and immune responses [102]. The excess secretion of CtsB by different types of cells present in TME including tumor cells, tumor-associated macrophages (TAM) and fibroblasts, makes them potent cancer-specific targets [103]. Mikhaylov et al. developed the liposomal nanocarrier (LNC) that contains CtsB inhibitor (NS-629) to target CtsB in the TME. NS-629 is linked to a PEG-functionalized lipid by a lipid linker to form LNC that can target CtsB on tumor (LNC-NS-629). They functionalized LNC-NS-629 by tagging the Gadolinium (Gd) to enhance the contrast at tumor sites when viewed under MRI. After MRI, they found that there was no undesirable accumulation of LNC-NS-629 in normal healthy tissues. Thus, Gd conjugated LNC-NS-629, can be used as a potential diagnostic tool. They also loaded LNC-NS-629 with Alexa Fluor 555 to confirm the target at tissue level when viewed under fluorescence microscopy. Upon examination, only TME cells showed fluorescent LNC-NS-629. Finally, when they encapsulated the anticancer drug doxorubicin in LNC-NS-629 to target primary MMTV-PyMT tumor cells, they found that it gaves 22-fold more increased death of tumor cells when compared to doxorubicin encapsulated in the LNC liposomes withoutNS-629 [104].

Besides the role of TAMs in tumor progression, invasion and metastasis, they can also be a potent drug target. The over-expression of mannose receptor on the TAMs was used for targeting TAM positive carcinomas [105]. Zhu et al. developed the PEG-sheddable PLGA NP conjugated with mannose as the ligand. PEG shedding was used to lessen the uptake of NPs by normal cells. They quantified the uptake amount (in vitro and in vivo fate) by labelling PLGA with FITC when viewed under a fluorescence microscope. This system may be used to target drugs like Yondelis and bisphosphonate clodronate to TAMs [90]. Various agents for Macrophage-specific PET imaging have been designed [106]. M1 and M2 macrophages of TAMs have opposite functions, where M1 shows antitumor activity and M2 is known to show pro-tumorigenic properties. Henceforth, recent focus shifted to the development of NP which can inhibit the M2 macrophages functions and convert them into M1 macrophages [107].

## 4. Efficient Drug Delivery Systems

Efficiency of drug delivery is the key element in improving the present status for both diagnosis and therapy of cancer. Multifunctional upconversion NPs (UCNPs) are used for effective cancer therapy. These NPs are used in nuclear targeting therapy where they deliver the anti-cancer drug into the cancer cell nucleus and also monitor the process using imaging modalities such as MRI, with the help of trans activator transcription (TAT) peptide. This is utilized as a cell penetrating peptide (CPP) derived from human immunodeficiency virus 1 (HIV-1) and thus, helps efficiently in the uptake of the NP [126]. The multifunctional UCNP drug delivery system carries the gene with anticancer drug (DOX), which replaces the mutated gene and also, induces the apoptosis of the tumor cell by damaging the DNA [127]. Here, we review the different types of drug delivery systems and nanoparticles which deliver the drug in an effective manner.

### 4.1. pH Based Drug Delivery System

Nanoparticles that are used particularly for targeting tumors have a drug releasing system which releases the drug when exposed to a particular pH. This system is based on the fact that the pH level of the tumor cells is much lower compared to that of normal cells. Griset et al. developed a paclitaxel-loaded polymeric nanoparticle that targets tumor cells and releases the drug when exposed to mild acidic condition. It was demonstrated that even at a low dose, the drug delivery of this nanoparticle was found to be highly efficient [128]. Furthermore, such a pH-dependent drug delivery system can be developed using graphene. Graphene-based nanomedicine, also called pH responsive chemotherapy, is used in various types of cancer treatments. Zheng et al. conducted a study to explore the efficiency of graphene NPs in both drug delivery as well as imaging probe. They inferred that these NPs have greater surface area which increases the amount of drug that can be loaded on a single NP [129].

Recent advancements of this pH based drug delivery system include pH responsive hyaluronic acid functionalized manganese dioxide nanosheets. These nanosheets can absorb cisplatin, a therapeutic drug which is delivered at the tumor target on acquisition of lower pH and reduced glutathione. The Mn^2+^ released in this process enables imaging using MRI thus providing a theranostic approach [130]. Aggregation induced emission (AIE) based fluorophores that show strong fluorescence and imaging abilities are considered better these days than the conventional fluorescent dyes which pose a disadvantage due to toxicity and fluorescence quenching. A multifunctional drug delivery system, MPEG-hyd-TPE was constructed by conjugating the AIE fluorophore tetraphenylethene (TPE) to MPEG (methyl polyethylene glycol) by a hydrazone bond which when it comes in contact with the low pH conditions, breaks the hydrazone bond and releases the doxorubicin drug. The cell imaging method employed is fluorescence microscopy, hence giving a theranostic application [131].

### 4.2. Enzyme-Responsive Silica Based Nanomedicine

The mesoporous silica nanoparticle (MSN) is another type of efficient drug delivery system that acts as a novel vehicle for the transport of anti-cancer drugs, which provides a large structural central cavity for drug encapsulation which in turn improves the rate of accumulation of the drug [132]. This silica-based NP is specifically designed for colonic conditions because the anti-cancer drug 5-fluoruracil (5FU) is a water-soluble drug activated by the colonic enzyme mixture present in the gastrointestinal (GI) track. Doxorubicin conjugated enzyme-cleavable precursors attached to silica coated magnetic nanoparticles by click chemistry, enables this NP moiety to release the drug efficiently when it comes in contact with cathepsin B which is usually overexpressed in cancer cells. The live imaging of drug release can be monitored using MRI and fluorescence [133]. Another similar study in which a nanoprobe containing hydroxylated mesoporus silica (HMNS) with polyethyleneamine (PEI) alongside Gadolinium (Gd) and Folic Acid (FA) was designed and doxorubicin, which is loaded in the mesoporous silica, released on pH control. This platform enables imaging using a magnetic resonance spectrometer and also to attain efficient drug delivery of doxorubicin to the tumor [134].

### 4.3. Liposome Mediated Drug

Liposome mediated nanoparticle drug delivery is non-toxic and non-immunogenic. It is mostly encapsulated in order to protect it from degradation. Liposome formulated nanostructures are the most widely used method for treatment of various types of cancer [135]. The use of liposomes to deliver anticancer drugs facilitates the protection of healthy sensitive tissue from the cytotoxic effects of the drug. Moreover, the circulation time of the drug can be increased by incorporating polyethylene glycol on the phospholipid bilayer of liposome which results in pegylated liposomes. These days, to increase the specificity of the liposome for targeting the desired tumor tissue, peptides and antibodies are used as targeting sites on the liposome [136]. Efficiency of drug delivery is also enhanced by modifying the drug to lipid ratio. This was observed when Johnston et al. conducted an experiment to study the effect of drug to lipid ratio (D/L) on the drug release rate. They showed the changes in releasing properties of vincristine encapsulated in large unilamellar vesicle (LUV) is regulated by various values of the D/L ratio. They concluded that doxorubicin loaded into this system (Lipo-Dox) at a D/L (*wt*/*wt*) ratio of 0.46 showed effective drug release [137]. Doxorubicin loaded liposomes have the advantage of delivering doxorubicin with reduced toxicity and prolonged circulation. Apart from this, other factors such as the structure of the liposome and the drug loading efficiency were evaluated by Z. Ali Mohammadi et al. The drug delivery efficiency and release rate was found to be maximum at pH 5.5, at a liposome composition containing unsaturated lipid and a sustained release was observed for liposome composition with cholesterol [138]. Chang et al. used another strategy to enhance efficiency by conjugating a peptide, SP5-2, to LipoDox which binds non-small cell lung cancer (NSCLC) cells and they observed a 11.2 fold improvement in the drug delivery efficiency compared to free doxorubicin [139]. Ze Lie et al. developed a system containing substrate of legumain Alanine-alanine-asparagine (AAN) and cell-penetrating peptides such as (TAT) This combination of AAN-TAT-liposome carrying the doxorubicin drug exhibited an increase in the delivery of doxorubicin to kill the tumor [140]. Another liposome composition containing paclitaxel called EndoTAG-1 is used to treat triple negative breast cancer and pancreatic cancer. This system of drug delivery along with DOTAP (1,2-dioleoyl-3-trimethylammonium propane) can be used to increase the affinity towards the tumor [141]. 

### 4.4. Polymeric Based Nanoparticle Theranostics

Polymeric based nanospheres, nanocapsules, and nanoparticles are made up of a biodegradable and biocompatible polymeric compound which encapsulates the drug inside the cavity. In the field of cancer theranostics, PNPs (Polymeric Nano Particles) serve as a promising vehicle for drug delivery because they carry the drug effectively to the specific organ. The nanometer size of the PNPs facilitate effective and easy transportation through the cell membrane [142] The polymers can degrade into safe components and pose lesser risk of toxicity. Examples of such polymers include polyethylene glycol, poly(dl-Lactic acid), poly(dl-glycolic acid) which are used for polymer based nanotheranostics. Along with this, an imaging agent is also incorporated such as an MRI contrast agent, radionuclide, fluorophore etc. This can further be enhanced by tagging a targeting ligand on the NP that specifically targets the tumor site [143]. A study by Napp et al. used polystyrene nanoparticles (PS-NPs) with palladium based tetraphenylporphyrin which has an emission wavelength in the NIR range. These PS-NPs with polyethylene glycol and a herceptin antibody bind to the HER2/neu-overexpressed in the tumor cell. This binding, revealed a unique ratiometric response in a hypoxic tumor cell. Thus, this enables a novel imaging strategy [144]. Incorporating a theranostic approach, an interesting study by Jain et al. involves the use of iron oxide NP along with a coating of oleic acid and amphiphilic block copolymer which maybe poly(ethylene oxide) (PEO) and poly(p-phenylene oxide) (PPO) that can deliver anticancer drugs (doxorubicin, paclitaxel) to a targeted tumor site. This nanoparticle system also has MRI properties that enable us to image the real time monitoring of drug distribution and response to chemotherapy on tumor progression [145]. Another interesting study was conducted by Gao et al. on the encapsulation of tadpole shaped polymer CDenPLA (cyclodextrin poly(dl-lactide)). This new copolymer nano-particle (377 nm size) carries and effectively (approx. 71.6%) releases the BSA (Bovine serum albumin) which was also confirmed using H NMR, SDS-PAGE and circular dichroism spectra [146].

## 5. Challenges in Effective Cancer Theranostics

One of the major challenges faced in nanotheranostics is the stability of the functionalization between the nanoprobe and the particular drug. Though this challenge can be overcome by modifying the surface of the nanoprobe, it gives rise to another problem—the control over the size of the nanostructure. This is because some applications, such as the probes for brain or liver cancers require a much smaller nanocarrier in order to efficiently penetrate the cells [147]. The efficiency of multifunctional nanocarriers is challenged by the specificity of cancer cell response as different types of cancer cells are responsive to different types of drugs. Similarly, some type of cancer cells can be resistant to particular drugs too. Thus, the study of multifunctional theranostic nanoprobes is not an easy feat in cancer theranostics [148]. However, to overcome the challenges of non-responsive cell receptor, the target may not be the cell itself. The NPs can be made sensitive to external factors of the TME such as pH or temperature [149]. If it is a pH-sensitive NP, it is preferred to be developed as an acidity-activated particle in cancer theranostics as the pH in the TME is usually low, indicating an acidic environment.

Nanotoxicity is an important factor in appraising the safety of the probe’s use in the future. It also proves to be a major concern in therapeutic techniques because negative interaction between NP and the drug might produce free radicals. This may in turn target the healthy neighboring cells instead of the cancer cells [150]. This phenomenon defeats the purpose of improving the patients’ health by killing the healthy cells. Some nanoprobes may remain in the body even after complete phagocytosis of cancer cells which also leads to nanotoxicity by which the patient may experience side effects. Silicon-based nanoprobes have been developed to overcome this obstacle but are still undergoing research as their speed of delivery appears inadequate [151]. However, the NPs designed particularly for neuroimaging have another challenge, which is the need for them to cross the selectively permeable BBB layer. There are not many probes which can overcome this problem. Thus, a lot of research experiments are being conducted in order to find out the transporter that can facilitate the crossing of the BBB layer [148].

In recent years, attaching immunosuppressors to the nanoprobes seems to have been highly welcomed as it eliminates the risk of triggering the immune system thus, possibly leading to autophagy [152]. Autophagy is a phenomenon where the immune cells, triggered by a foreign substance invasion attack the healthy cells along with the foreign substance which in turn disturbs the metabolic homeostasis [105].

Radioactive imaging probes have an attribute called half-life, which denotes the amount of time they remain active. Usually, the probes have low half-time denoting their poor-viability and thus demanding a faster imaging process [153]. Hence, multifunctional nanocarriers such as liposome and micelles are mostly used for imaging and drug delivery as they have the ability to reduce the side effects, lower the toxicity, and increase the uptake of the drug by tumor cells efficiently.

## 6. Conclusions

It has been a milestone in cancer research to observe the paradigm shift of cancer cells from an isolated mass of cells displaying uncontrolled division to one that undergoes complicated communication with the TME. Since then, many studies have expanded our knowledge on the different components of TME and their roles in tumor development. The rapid advancements in nanotechnology allow nanotheranostics to be implemented in the combat against cancer.

The abnormal pH, IFP, and oxygen concentration in the TME serve as a suitable target for nanoprobes for cancer detection. Moreover, the difference in the environment of healthy cells and TME allows researchers to design drug carriers that release anticancer drugs in the TME. However, many of these drug carriers and molecular probes are still confined to the research domain and have yet to be transferred to a clinical setting. This review discussed the methods with which different elements in TME are targeted using nanotheranostics for detection, therapy and drug design in cancer, for example, the tumor-associated stromal cells (TASCs) that consist of tumor-associated macrophages and cancer-associated fibroblast. The overexpression of genes in TASCs poses a potential nanotheranostic target. Abnormally high level of lymphangiogenesis with overexpression of antigens is also another target for nanotheranostics. Apart from that, scientists have developed different nanoprobes to detect angiogenesis and the proteins attached to the newly made blood vessels using modalities like PET and MR imaging. Besides that, the signaling molecules and growth factors supplied by the ECM for tumor progression have provided potent cancer-specific target nanoprobe development. Lastly, we highlighted different drug delivery systems designed based on pH, enzyme, liposome, and topology. The shortcomings of these drug delivery systems, such as cytotoxicity and instability of functionalization between the drug and the NPs were discussed.

In summary, the rapid knowledge advancement in tumor microenvironment and its role in tumor development proffer researchers a better foundation to conduct cancer research. The development of TME-specific nanotheranostics will provide a faster and more accurate cancer diagnosis and therapy in the future, if it is translated in the clinical setting.

## 7. Future Perspective

As the role of TME in cancer progression becomes established, the future direction is to develop TME-targeted therapies and drug delivery systems to halt or slow down cancer malignancy. Although numerous studies have highlighted TME-specific cancer treatment, there are challenges that are still unresolved. These include the dynamic changes in TME, heterogeneity of TME, and the complex nature of different molecular factors in TME. In addition, many studies discuss the interplay between a TME element and cancer growth. It is important to look into the network of all TME elements to elucidate the crosstalk between cancer growth and TME as a whole. Another area of interest is to establish the TME network based on specific types of cancer. For example, the TME of hepatocellular carcinoma and prostate cancer is different. Based on this difference, more specific and effective diagnostic or therapeutic methods can be developed.

In the near future, once the TME of a particular type of cancer has been established, personalized and targeted treatments can be tailored to replace non-specific chemotherapy or radiotherapy. Besides, understanding the cancer-TME interplay will help us look into therapies to reduce the recurrence of cancer. By doing so, there will be less societal and governmental burden imposed. Cancer progression can be controlled and monitored using TME-targeted therapies and diagnostics.

## Figures and Tables

**Table 1:** Significant probes incorporated for therapy and imaging of the tumor microenvironment.

Serial Number	Tumor Microenvironment	Imaging Modality	Probe Design	In Vivo Model	References
1	Hypoxia (deoxygenated cells)	PET	^18^F-FMISO	Glioblastoma patients	Spence et al. [24], Masaki et al. [108]
				Nine-week-old male BALB/c athymic nude mice	
2	Hypoxia (deoxygenated cells)	PET	^61^Cu-ATSM	Head and neck squamous cell carcinoma	Flynn et al. [25], Lapi et al. [109]
3	Hypoxia (deoxygenated cells)	MRS	2-nitro-α-[(2,2,2-trifluoroethoxy) methyl]-imidazole-1-ethanol	Gastrointestinal cancer mouse model	Procissi et al. [29], Papadopoulou et al. [110]
				BALB/c female Mice	
4	Hypoxia (deoxygenated cells)	MRS	GdDO3NI	Rat prostate cancer	Gulaka et al. [31]
5	Hypoxia (deoxygenated cells)	Optical Imaging	azo based fluorescent probe	Xenograft models	Kakkad et al. [33], Q. Cai et al. [111]
				In-vitro HeLA cells	
6	Hypoxia	Photoacoustic imaging	CD44v6-GNS	Xenografted mouse models of gastric cancer	Liang et al. [35]
7	pH	MRS	(imidazol-1-yl)3-ethyoxycarbonylpropionic acid	Human breast cancer cells (MCF-7 and mdamb-435), grown in the mammary fat pad of severe combined immunodeficient (SCID) mice.	Van Sluis et al. [112]
8	pH	MRS	2-(imidazol-1-yl) succinic acid	Gliomas in rat brain	Provent et al. [40]
9	pH	Optical	DilR-783-S	MDA-MB-435 xenograft model	L. Wang et al. [43]
10	pH	Optical	CEST with iopromide contrast agent	Breast cancer xenograft	Chen et al. [44], Moon et al. [113]
				Xenograft tumor of Raji lymphoma and five mice with a xenograft tumor of MCF-7 breast cancer	
11	pH	Optical	doxorubicin polymeric micelles	B16F10 tumor-bearing mice	Ko et al. [47], D Kim et al. [114]
				4–6 week old female nude mice (BALB/c nu/nu mice)	
12	pH	Photoacoustic Imaging	GNS-pHLIP	Xenograft models of gastric cancer	Tian et al. [48]
13	pH	Photoacoustic Imaging	BODPA-NP	HCT116 mice cell	Shi et al. [49]
14	Interstitial Fluid Pressure	MRI	Gd-DTPA	Xenograft mouse models	Hompland et al. [51], L J. Liu et al. [52]
				A-07 human melanoma xenografts growing in female BALB/c nu/nu mice	
15	Interstitial Fluid Pressure	Optical	Lysosome-Imatinib	BB16 melanoma mouse model	Fan et al. [53]
16	Tumor Vasculature	MRI	Photofrin iron oxide coated NPs	Rat glioma	Reddy et al. [55]
17	Tumor Vasculature	MRI	α5β1 RGD (Radiolabeled Arg Gly Asp) rhodamine nano particles	Α5β1 integrin in MDA-MD 435 xenograft mouse model	Schmieder et al. [57], Marelli et al. [115]
18	Tumor Vasculature	MRI	α5β1(ανβ3) fumagillin	Α5β1(ανβ3) in MDA MD 435 xenograft mouse model	Schmieder et al. [57]
19	Tumor Vasculature	MRI	NCAM targeted liposomes with doxorubicin and Gd	SCID male mice	Grange et al. [58]
20	Tumor Vasculature	MRI	PLP-LCL	B16.F10 melanoma cells injected to Male C57BI6 mice	Cittadino et al. [59]
21	Tumor Vasculature	MRI	SPIO magnetic nano particles	EGFP transfected U87MG human glioblastoma into SCID mouse	Fu Aihua et al. [56]
22	Tumor Vasculature	PET	^64^Cu-NOTA-RGO-TRC105	xenograft U87MG tumor-bearing mice	S. Shi et al. [65]
23	Tumor Vasculature	ImmunoPET	^124^I MORAb-004	Mice bearing MS1-hTEM1/fLuc or MS1/fLuc angioma grafts	Li et al. [68]
24	Tumor Vasculature	Photoacoustic imaging	PEG-CuS-NP	4T1 breast cancer tumor bearing mice	Zhou et al. [69]
25	VEGF-A	PET	^89^Zr bevacizumab	Human adenocarcinoma patients	Gaykema et al. [70], Golestani et al. [116]
				Human carotid endarterectomy (CEA) specimens.	
26	VEGF-A	PET	^124^I-VG67e	HT1080-26.6-bearing mice	Collingridge et al. [60]
27	VEGFR	PET	^64^Cu-DOTA-VEGF_121_	Mice bearing U87MG human glioblastomas	Ferrara 2009 [61], K. Chen et al. [117]
28	VEGFR	PET	NOTA-GO-VEGF121	4T1 murine breast tumors	Shi et al. [66], S Shi et al. [118]
29	VEGFR	SPECT	99mTc-scVEGF	Male Swiss–Webster mice	Levashova et al. [63]
30	VEGFR	Optical	VEGF121-Avi-streptavdidn IRDye800scVEGF/Cy	Mice bearing VEGFR-2–expressing 67NR murine breast tumors	Kang et al. [119]
31	ED-B of fibronectin	PET	^124^-L19-SIP	Xenograft nude mice	Tijink et al. [62]
32	Fibronectin	SPECT/CT	^123^I-L19(scFv)_2_	5 male patients with head and neck cancer.	Birchler et al. [71], Santimaria et al. [120]
				Twenty patients (34–79 years of age) with lung, colorectal, or brain cancer	
33	Lymphatic System	Optical	LyP-1-maleimide-PEG-PLGA-FTIC	Lymphatic metastasis tumor models, Nude BALB/c nu/nu mice	Luo et al. [78]
34	Lymphatic System	Optical	LyP-1-Cy5.5	4T1 murine breast cancer in mouse	Zhang et al. [79], Zhang et al. [121]
35	Lymphatic System	Optical	LyP-1-PM-ART	Nude mice bearing orthotopic MDA-MB-435S breast tumors	Z. Wang et al. [80]
36	Extracellular Matrix	Optical	HA-Au NPs-Fluorophore	C57BL/6 male mice were given a bolus injection of saline or of MMC formulations	Peer and Margalit [81], S. Lee et al. [122], H Lee et al. [123]
				Mice bearing SCC7 tumors	
				4-Week-old DBA-1J mice	
37	Extracellular Matrix	Optical	LOX antibody-copolymer	Mice bearing 4T1 tumors implanted within the mammary fat pad	Kanapathipillai et al. [82]
38	Extracellular Marix	PET	^64^Cu-DOTA-antiperiostin-F(ab′)2	Genetically engineered esophageal squamous cell carcinoma mouse models	Heidari et al. [87]
39	MMP-2	Optical	T7 peptide-LMWH-QD-LMWP-Fluorophore	Xenograft, ex vivo and in vivo of mice bearing HT1080 tumor	Y. Wang et al. [84]
40	Collagen	Optical	CMP-IR-Ahx-(GPO)9	Prostate cancer cells were implanted subcutaneously in non-obese diabetic (NOD)/severe-combined immunodeficient (SCID) mice.	Y. Li et al. [86], Y. Li et al. [124]
				SKH-1, DR-1 nude mice	
41	Stromal Cells (FAP-α)	Optical	ferritin-fluorescence peptide	Co-implants Mice bearing CAFs and PC-3 co implants	Ji et al. [100,101]
			Fibroblast activation protein α–specific, near-infrared peptide probe (KGPGPNQC) linked to Cy5.5 and a quencher dye, QSY21	Mice bearing C6 cell tumors (controls) or U87MG cell tumors	[125]
42	Stromal Cells (FAP-α)	Optical	CAP-doxorubicin-Nanoparticles	Mice bearing CAFs and PC-3 co implants	Ji et al. [100,101]
43	Stromal Cells (CtsB-PyMT tumor cells)	Optical	Doxorubicin-LNC-NS629-Gd-Alexa Fluor 555	Mice bearing orthotopically transplanted congenic mammary tumors	Mikhaylov et al. [104]
44	Stromal Cells (CtsB-PyMT tumor cells)	Optical	mannose-PLGA-FITC	C57BL/6 miceequation	Zhu et al. [90]
45	Stromal Cells (Podoplanin)	MRI	PEG-GoldMag-nanoparticles-PodAb	Rat breast tumor model	Yang et al. [76]

## References

[1] Swartz M.A., Iida N., Roberts E.W., Sangaletti S., Wong M.H., Yull F.E., Coussens L.M., DeClerck Y.A. (2012). Tumor microenvironment complexity: Emerging roles in cancer therapy. Cancer Res..

[2] Korneev K.V., Atretkhany K.-S.N., Drutskaya M.S., Grivennikov S.I., Kuprash D.V., Nedospasov S.A. (2017). TLR-signaling and proinflammatory cytokines as drivers of tumorigenesis. Cytokine.

[3] Spill F., Reynolds D.S., Kamm R.D., Zaman M.H. (2016). Impact of the physical microenvironment on tumor progression and metastasis. Curr. Opin. Biotechnol..

[4] Wu Y., Zhang W., Li J., Zhang Y. (2013). Optical imaging of tumor microenvironment. Am. J. Nucl. Med. Mol. Imaging.

[5] Thiruppathi R., Mishra S., Ganapathy M., Padmanabhan P., Gulyás B. (2017). Nanoparticle Functionalization and Its Potentials for Molecular Imaging. Adv. Sci..

[6] Haigron P., Dillenseger J.-L., Luo L., Coatrieux J.-L. (2010). Image-Guided Therapy: Evolution and Breakthrough. IEEE Eng. Med. Biol. Mag..

[7] Jo S.D., Ku S.H., Won Y.-Y., Kim S.H., Kwon I.C. (2016). Targeted Nanotheranostics for Future Personalized Medicine: Recent Progress in Cancer Therapy. Theranostics.

[8] Lee S., Xie J., Chen X. (2010). Peptide-based probes for targeted molecular imaging. Biochemistry.

[9] O’Shannessy D.J., Somers E.B., Albone E., Cheng X., Park Y.C., Tomkowicz B.E., Hamuro Y., Kohl T.O., Forsyth T.M., Smale R. (2011). Characterization of the human folate receptor α via novel antibody-based probes. Oncotarget.

[10] Truong N.P., Whittaker M.R., Mak C.W., Davis T.P. (2015). The importance of nanoparticle shape in cancer drug delivery. Expert Opin. Drug Deliv..

[11] Banerjee A., Qi J., Gogoi R., Wong J., Mitragotri S. (2016). Role of nanoparticle size, shape and surface chemistry in oral drug delivery. J. Control. Release.

[12] Loverde S.M., Klein M.L., Discher D.E. (2012). Nanoparticle shape improves delivery: Rational coarse grain molecular dynamics (rCG-MD) of taxol in worm-like PEG-PCL micelles. Adv. Mater..

[13] Kim J.B., Park K., Ryu J., Lee J.J., Lee M.W., Cho H.S., Nam H.S., Park O.K., Song J.W., Kim T.S. (2016). Intravascular optical imaging of high-risk plaques in vivo by targeting macrophage mannose receptors. Sci. Rep..

[14] Xie T., Dong B., Yan Y., Hu G., Xu Y. (2016). Association between MMP-2 expression and prostate cancer: A meta-analysis. Biomed. Rep..

[15] Yu M.K., Park J., Jon S. (2012). Targeting Strategies for Multifunctional Nanoparticles in Cancer Imaging and Therapy. Theranostics.

[16] Chen F., Zhuang X., Lin L., Yu P., Wang Y., Shi Y., Hu G., Sun Y. (2015). New horizons in tumor microenvironment biology: Challenges and opportunities. BMC Med..

[17] Thomlinson R.H., Gray L.H. (1955). The Histological Structure of Some Human Lung Cancers and the Possible Implications for Radiotherapy. Br. J. Cancer.

[18] Chen Y.-Q., Zhao C.-L., Li W. (2009). Effect of hypoxia-inducible factor-1α on transcription of survivin in non-small cell lung cancer. J. Exp. Clin. Cancer Res..

[19] Bussink J., Kaanders J.H., Rijken P.F., Raleigh J.A., Van der Kogel A.J. (2000). Changes in Blood Perfusion and Hypoxia after Irradiation of a Human Squamous Cell Carcinoma Xenograft Tumor Line. Radiat. Res..

[20] Erler J.T., Bennewith K.L., Nicolau M., Dornhöfer N., Kong C., Le Q.-T., Chi J.-T.A., Jeffrey S.S., Giaccia A.J. (2006). Lysyl oxidase is essential for hypoxia-induced metastasis. Nature.

[21] Michieli P. (2009). Hypoxia, angiogenesis and cancer therapy: To breathe or not to breathe?. Cell Cycle.

[22] Semenza G. (2009). HIF-1 Inhibitors for Cancer Therapy: From Gene Expression to Drug Discovery. Curr. Pharm. Des..

[23] Penet M.-F., Krishnamachary B., Chen Z., Jin J., Bhujwalla Z.M. (2014). Molecular Imaging of the Tumor Microenvironment for Precision Medicine and Theranostics. Adv. Cancer Res..

[24] Spence A.M., Muzi M., Swanson K.R., Sullivan F.O., Jason K., Rajendran J.G., Adamsen T.C.H., Link J.M., Swanson P.E., Yagle K.J. (2015). Radiotherapy: Correlation with Time to Progression and Survival. Clin. Cancer Res..

[25] Flynn R.T., Bowen S.R., Bentzen S.M., Mackie T.R., Jeraj R. (2008). Intensity modulated X-ray (IMXT) vs. proton (IMPT) therapy for theragnostic hypoxia-based dose painting. Phys. Med. Biol..

[26] Carlin S., Humm J.L. (2012). PET of hypoxia: Current and future perspectives. J. Nucl. Med..

[27] Mason R.P., Shukla H., Antich P.P. (1993). In Vivo oxygen tension and temperature: Simultaneous determination using19F NMR spectroscopy of perfluorocarbon. Magn. Reson. Med..

[28] Kodibagkar V.D., Cui W., Merritt M.E., Mason R.P. (2006). Novel 1H NMR approach to quantitative tissue oximetry using hexamethyldisiloxane. Magn. Reson. Med..

[29] Procissi D., Claus F., Burgman P., Koziorowski J., Chapman J.D., Thakur S.B., Matei C., Ling C.C., Koutcher J.A. (2007). In Vivo 19F magnetic resonance spectroscopy and chemical shift imaging of tri-fluoro-nitroimidazole as a potential hypoxia reporter in solid tumors. Clin. Cancer Res..

[30] Lee C.P., Payne G.S., Oregioni A., Ruddle R., Tan S., Raynaud F.I., Eaton D., Campbell M.J., Cross K., Halbert G. (2009). A phase I study of the nitroimidazole hypoxia marker SR4554 using 19F magnetic resonance spectroscopy. Br. J. Cancer.

[31] Gulaka P.K., Rojas-Quijano F., Kovacs Z., Mason R.P., Sherry A.D., Kodibagkar V.D. (2014). GdDO3NI, a nitroimidazole-based T1 MRI contrast agent for imaging tumor hypoxia in vivo. JBIC J. Biol. Inorg. Chem..

[32] Raman V., Artemov D., Pathak A.P., Winnard P.T., McNutt S., Yudina A., Bogdanov A., Bhujwalla Z.M. (2006). Characterizing vascular parameters in hypoxic regions: A combined magnetic resonance and optical imaging study of a human prostate cancer model. Cancer Res..

[33] Kakkad S.M., Solaiyappan M., Argani P., Sukumar S., Jacobs L.K., Leibfritz D., Bhujwalla Z.M., Glunde K. (2012). Collagen I fiber density increases in lymph node positive breast cancers: Pilot study. J. Biomed. Opt..

[34] Xia J., Yao J., Wang L.V. (2014). Photoacoustic tomography: Principles and advances. Electromagn. Waves.

[35] Liang S., Li C., Zhang C., Chen Y., Xu L., Bao C., Wang X., Liu G., Zhang F., Cui D. (2015). CD44v6 monoclonal antibody-conjugated gold nanostars for targeted photoacoustic imaging and plasmonic photothermal therapy of gastric cancer stem-like cells. Theranostics.

[36] Konishi M., Kawamoto K., Izumikawa M., Kuriyama H., Yamashita T. (2008). Gene transfer into guinea pig cochlea using adeno-associated virus vectors. J. Gene Med..

[37] Stock C., Schwab A. (2009). Protons make tumor cells move like clockwork. Pflugers Arch. Eur. J. Physiol..

[38] Gillies R.J., Robey I., Gatenby R.A. (2008). Causes and consequences of increased glucose metabolism of cancers. J. Nucl. Med..

[39] Robey I.F., Baggett B.K., Kirkpatrick N.D., Roe D.J., Dosescu J., Sloane B.F., Hashim A.I., Morse D.L., Raghunand N., Gatemnby R.A. (2009). Bicarbonate increases tumor pH and inhibits spontaneous metastases. Cancer Res..

[40] Provent P., Benito M., Hiba B., Farion R., López-Larrubia P., Ballesteros P., Rémy C., Segebarth C., Cerdán S., Coles J.A. (2007). Serial in vivo spectroscopic nuclear magnetic resonance imaging of lactate and extracellular pH in rat gliomas shows redistribution of protons away from sites of glycolysis. Cancer Res..

[41] Kalash R., Berhane H., Au J., Rhieu B.H., Epperly M.W., Goff J., Dixon T., Wang H., Zhang X., Franicola D. (2014). Differences in irradiated lung gene transcription between fibrosis-prone C57BL/6NHsd and fibrosis-resistant C3H/HeNHsd mice. In Vivo.

[42] Moon S.-H., Yang B.Y., Kim Y.J., Hong M.K., Lee Y.-S., Lee D.S., Chung J.-K., Jeong J.M. (2016). Development of a complementary PET/MR dual-modal imaging probe for targeting prostate-specific membrane antigen (PSMA). Nanomed. Nanotechnol. Biol. Med..

[43] Wang L., Zhu X., Xie C., Ding N., Weng X., Lu W., Wei X., Li C. (2012). Imaging acidosis in tumors using a pH-activated near-infrared fluorescence probe. Chem. Commun..

[44] Chen L.Q., Howison C.M., Jeffery J.J., Robey I.F., Kuo P.H., Pagel M.D. (2014). Evaluations of extracellular pH within in vivo tumors using acidoCEST MRI. Magn. Reson. Med..

[45] Gallagher F.A., Kettunen M.I., Day S.E., Hu D.E., Ardenkjaer-Larsen J.H., Zandt R.i., Jensen P.R., Karlsson M., Golman K., Lerche M.H. (2008). Magnetic resonance imaging of pH in vivo using hyperpolarized 13C-labelled bicarbonate. Nature.

[46] Gao G.H., Li Y., Lee D.S. (2013). Environmental pH-sensitive polymeric micelles for cancer diagnosis and targeted therapy. J. Control. Release.

[47] Ko J., Park K., Kim Y.S., Kim M.S., Han J.K., Kim K., Park R.W., Kim I.S., Song H.K., Lee D.S. (2007). Tumoral acidic extracellular pH targeting of pH-responsive MPEG-poly(β-amino ester) block copolymer micelles for cancer therapy. J. Control. Release.

[48] Tian Y., Zhang Y., Teng Z., Tian W., Luo S., Kong X., Su X., Tang Y., Wang S., Lu G. (2017). pH-Dependent Transmembrane Activity of Peptide-functionalized Gold Nanostars for Computed Tomography/Photoacoustic Imaging and Photothermal Therapy. ACS Appl. Mater. Interfaces.

[49] Shi B., Gu X., Fei Q., Zhao C. (2017). Photoacoustic probes for real-time tracking of endogenous H_2_S in living mice. Chem. Sci..

[50] Siegler E.L., Kim Y.J., Wang P. (2016). Nanomedicine targeting the tumor microenvironment: Therapeutic strategies to inhibit angiogenesis, remodel matrix, and modulate immune responses. J. Cell. Immunother..

[51] Hompland T., Ellingsen C., Øvrebø K.M., Rofstad E.K. (2012). Interstitial fluid pressure and associated lymph node metastasis revealed in tumors by dynamic contrast-enhanced MRI. Cancer Res..

[52] Liu L.J., Brown S.L., Ewing J.R., Ala B.D., Schneider K.M., Schlesinger M. (2016). Estimation of Tumor Interstitial Fluid Pressure (TIFP) Noninvasively. PLoS ONE.

[53] Fan Y., Du W., He B., Fu F., Yuan L., Wu H., Dai W., Zhang H., Wang X., Wang J. (2013). The reduction of tumor interstitial fluid pressure by liposomal imatinib and its effect on combination therapy with liposomal doxorubicin. Biomaterials.

[54] Lubberink M., Golla S.S.V., Jonasson M., Rubin K., Glimelius B., Sorensen J., Nygren P. (2015). ^15^O-Water PET Study of the Effect of Imatinib, a Selective Platelet-Derived Growth Factor Receptor Inhibitor, Versus Anakinra, an IL-1R Antagonist, on Water-Perfusable Tissue Fraction in Colorectal Cancer Metastases. J. Nucl. Med..

[55] Reddy G.R., Bhojani M.S., McConville P., Moody J., Moffat B.A., Hall D.E., Kim G., Koo Y.E.L., Woolliscroft M.J., Sugai J.V. (2006). Vascular targeted nanoparticles for imaging and treatment of brain tumors. Clin. Cancer Res..

[56] Nanoparticles F.M., Enhanced M., Imaging C., Subjects L. (2012). Fluorescent Magnetic Nanoparticles for Magnetically Enhanced Cancer Imaging and Targeting in Living. ASC Nano.

[57] Schmieder A.H., Caruthers S.D., Zhang H., Williams T.A., Robertson J.D., Wickline S.A., Lanza G.M. (2008). Three-dimensional MR mapping of angiogenesis with α_5_β_1_(α_ν_β_3_)-targeted theranostic nanoparticles in the MDA-MB-435 xenograft mouse model. FASEB J..

[58] Grange C., Geninatti-Crich S., Esposito G., Alberti D., Tei L., Bussolati B., Aime S., Camussi G. (2010). Combined delivery and magnetic resonance imaging of neural cell adhesion molecule-targeted doxorubicin-containing liposomes in experimentally induced Kaposi’s sarcoma. Cancer Res..

[59] Cittadino E., Ferraretto M., Torres E., Maiocchi A., Crielaard B.J., Lammers T., Storm G., Aime S., Terreno E. (2012). MRI evaluation of the antitumor activity of paramagnetic liposomes loaded with prednisolone phosphate. Eur. J. Pharm. Sci..

[60] Collingridge D.R., Carroll V.A., Glaser M., Aboagye E.O., Osman S., Hutchinson O.C., Barthel H., Luthra S.K., Brady F., Bicknell R. (2002). The development of [124I]iodinated-VG76e: A novel tracer for imaging vascular endothelial growth factor in vivo using positron emission tomography. Cancer Res..

[61] Ferrara N. (2009). Vascular endothelial growth factor. Arterioscler. Thromb. Vasc. Biol..

[62] Tijink B.M., Perk L.R., Budde M., Stigter-Van Walsum M., Visser G.W.M., Kloet R.W., Dinkelborg L.M., Leemans C.R., Neri D., Van Dongen G.A.M.S. (2009). 124I-L19-SIP for immuno-PET imaging of tumour vasculature and guidance of 131I-L19-SIP radioimmunotherapy. Eur. J. Nucl. Med. Mol. Imaging.

[63] Levashova Z., Backer M., Backer J.M., Blankenberg F.G. (2009). Imaging vascular endothelial growth factor (VEGF) receptors in turpentine-induced sterile thigh abscesses with radiolabeled single-chain VEGF. J. Nucl. Med..

[64] Leung K. (2004). Biotinylated Vascular Endothelial Growth Factor_121_-Avi-Streptavidin-IRDye800.

[65] Shi S., Yang K., Hong H., Valdovinos H.F., Nayak T.R., Zhang Y., Theuer C.P., Barnhart T.E., Liu Z., Cai W. (2013). Tumor vasculature targeting and imaging in living mice with reduced graphene oxide. Biomaterials.

[66] Shi S., Yang K., Hong H., Nickles R., Liu Z., Cai W. (2014). In Vivo tumor vasculature targeting and imaging with VEGF-conjugated nanographene oxide. J. Nucl. Med..

[67] Chen F., Nayak T.R., Goel S., Valdovinos H.F., Hong H., Theuer C.P., Barnhart T.E., Cai W. (2014). In Vivo tumor vasculature targeted PET/NIRF imaging with TRC105 (Fab)-conjugated, dual-labeled mesoporous silica nanoparticles. Mol. Pharm..

[68] Li C., Chacko A.-M., Hu J., Hasegawa K., Swails J., Grasso L., El-Deiry W.S., Nicolaides N., Muzykantov V.R., Divgi C.R. (2014). Antibody-based tumor vascular theranostics targeting endosialin/TEM1 in a new mouse tumor vascular model. Cancer Biol. Ther..

[69] Zhou M., Ku G., Pageon L., Li C. (2014). Theranostic probe for simultaneous in vivo photoacoustic imaging and confined photothermolysis by pulsed laser at 1064 nm in 4T1 breast cancer model. Nanoscale.

[70] Gaykema S.B.M., Brouwers A.H., Lub-de Hooge M.N., Pleijhuis R.G., Timmer-Bosscha H., Pot L., van Dam G.M., van der Meulen S.B., de Jong J.R., Bart J. (2013). 89Zr-Bevacizumab PET Imaging in Primary Breast Cancer. J. Nucl. Med..

[71] Birchler M.T., Thuerl C., Schmid D., Neri D., Waibel R., Schubiger A., Stoeckli S.J., Schmid S., Goerres G.W. (2007). Immunoscintigraphy of patients with head and neck carcinomas, with an anti-angiogenetic antibody fragment. Otolaryngol. Head Neck Surg..

[72] Shayan R., Achen M.G., Stacker S.A. (2006). Lymphatic vessels in cancer metastasis: Bridging the gaps. Carcinogenesis.

[73] Bhang S.H., Won N., Lee T.-J., Jin H., Nam J., Park J., Chung H., Park H.-S., Sung Y.-E., Hahn S.K. (2009). Hyaluronic acid-quantum dot conjugates for in vivo lymphatic vessel imaging. ACS Nano.

[74] Mumprecht V., Honer M., Vigl B., Proulx S.T., Trachsel E., Kaspar M., Banziger-Tobler N.E., Schibli R., Neri D., Detmar M. (2010). In Vivo Imaging of inflammation-and tumor-induced lymph node lymphangiogenesis by immuno--positron emission tomography. Cancer Res..

[75] Yousefi S., Zhi Z., Wang R.K. (2014). Label-free optical imaging of lymphatic vessels within tissue beds in vivo. IEEE J. Sel. Top. Quantum Electron..

[76] Yang H., Zou L.G., Zhang S., Gong M.F., Zhang D., Qi Y.Y., Zhou S.W., Diao X.W. (2013). Feasibility of MR imaging in evaluating breast cancer lymphangiogenesis using Polyethylene glycol-GoldMag nanoparticles. Clin. Radiol..

[77] Sun L., Wu Q., Peng F., Liu L., Gong C. (2015). Strategies of polymeric nanoparticles for enhanced internalization in cancer therapy. Colloids Surf. B Biointerfaces.

[78] Luo G., Yu X., Jin C., Yang F., Fu D., Long J., Xu J., Zhan C., Lu W. (2010). LyP-1-conjugated nanoparticles for targeting drug delivery to lymphatic metastatic tumors. Int. J. Pharm..

[79] Zhang F., Niu G., Lin X., Jacobson O., Ma Y., Eden H.S., He Y., Lu G., Chen X. (2012). Imaging tumor-induced sentinel lymph node lymphangiogenesis with LyP-1 peptide. Amino Acids.

[80] Wang Z., Yu Y., Ma J., Zhang H., Zhang H., Wang X., Wang J., Zhang X., Zhang Q. (2012). LyP-1 modification to enhance delivery of artemisinin or fluorescent probe loaded polymeric micelles to highly metastatic tumor and its lymphatics. Mol. Pharm..

[81] Peer D., Margalit R. (2004). Loading mitomycin C inside long circulating hyaluronan targeted nano-liposomes increases its antitumor activity in three mice tumor models. Int. J. Cancer.

[82] Kanapathipillai M., Mammoto A., Mammoto T., Kang J.H., Jiang E., Ghosh K., Korin N., Gibbs A., Mannix R., Ingber D.E. (2012). Inhibition of mammary tumor growth using lysyl oxidase-targeting nanoparticles to modify extracellular matrix. Nano Lett..

[83] Yhee J.Y., Kim S.A., Koo H., Son S., Ryu J.H., Youn I.C., Choi K., Kim K. (2012). Optical imaging of cancer-related proteases using near-infrared fluorescence matrix metalloproteinase-sensitive and cathepsin B-sensitive probes. Theranostics.

[84] Wang Y., Lin T., Zhang W., Jiang Y., Jin H., He H., Yang V.C., Chen Y., Huang Y. (2015). A prodrug-type, MMP-2-targeting nanoprobe for tumor detection and imaging. Theranostics.

[85] Schuerle S., Dudani J.S., Christiansen M.G., Anikeeva P., Bhatia S.N. (2016). Magnetically Actuated Protease Sensors for in vivo Tumor Profiling. Nano Lett..

[86] Li Y., Foss C.A., Summerfield D.D., Doyle J.J., Torok C.M., Dietz H.C., Pomper M.G., Yu S.M. (2012). Targeting collagen strands by photo-triggered triple-helix hybridization. Proc. Natl. Acad. Sci. USA.

[87] Heidari P., Esfahani S.A., Turker N.S., Wong G., Wang T.C., Rustgi A.K., Mahmood U. (2015). Imaging of secreted extracellular periostin, an important marker of invasion in the tumor microenvironment in esophageal cancer. J. Nucl. Med..

[88] Mao Y., Keller E.T., Garfield D.H., Shen K., Wang J. (2013). Stromal cells in tumor microenvironment and breast cancer. Cancer Metastasis Rev..

[89] Mantovani A., Bottazzi B., Colotta F., Sozzani S., Ruco L. (1992). The origin and function of tumor-associated macrophages. Immunol. Today.

[90] Zhu S., Niu M., O’Mary H., Cui Z. (2013). Targeting of tumor-associated macrophages made possible by PEG-sheddable, mannose-modified nanoparticles. Mol. Pharm..

[91] Östman A., Augsten M. (2009). Cancer-associated fibroblasts and tumor growth—Bystanders turning into key players. Curr. Opin. Genet. Dev..

[92] Kalluri R., Zeisberg M. (2006). Fibroblasts in cancer. Nat. Rev. Cancer.

[93] Aggarwal S., Brennen W.N., Kole T.P., Schneider E., Topaloglu O., Yates M., Cotter R.J., Denmeade S.R. (2008). Fibroblast activation protein peptide substrates identified from human collagen I derived gelatin cleavage sites. Biochemistry.

[94] Brennen W.N., Isaacs J.T., Denmeade S.R. (2012). Rationale behind targeting fibroblast activation protein—Expressing carcinoma-associated fibroblasts as a novel chemotherapeutic strategy. Mol. Cancer Ther..

[95] Granot D., Addadi Y., Kalchenko V., Harmelin A., Kunz-Schughart L.A., Neeman M. (2007). In Vivo imaging of the systemic recruitment of fibroblasts to the angiogenic rim of ovarian carcinoma tumors. Cancer Res..

[96] Loeffler M., Krüger J.A., Niethammer A.G., Reisfeld R.A. (2006). Targeting tumor-associated fibroblasts improves cancer chemotherapy by increasing intratumoral drug uptake. J. Clin. Investig..

[97] Miao L., Huang L. (2015). Exploring the tumor microenvironment with nanoparticles. Nanotechnology-Based Precision Tools for the Detection and Treatment of Cancer.

[98] Milner J.M., Patel A., Rowan A.D. (2008). Emerging roles of serine proteinases in tissue turnover in arthritis. Arthritis Rheumatol..

[99] Kennedy A., Dong H., Chen D., Chen W.-T. (2009). Elevation of seprase expression and promotion of an invasive phenotype by collagenous matrices in ovarian tumor cells. Int. J. Cancer.

[100] Ji T., Zhao Y., Wang J., Zheng X., Tian Y., Zhao Y., Nie G. (2013). Tumor Fibroblast Specific Activation of a Hybrid Ferritin Nanocage-Based Optical Probe for Tumor Microenvironment Imaging. Small.

[101] Ji T., Zhao Y., Ding Y., Wang J., Zhao R., Lang J., Qin H., Liu X., Shi J., Tao N. (2016). Transformable Peptide Nanocarriers for Expeditious Drug Release and Effective Cancer Therapy via Cancer-Associated Fibroblast Activation. Angew. Chem..

[102] Gondi C.S., Rao J.S. (2013). Cathepsin B as a cancer target. Expert Opin. Ther. Targets.

[103] Victor B.C., Anbalagan A., Mohamed M.M., Sloane B.F., Cavallo-Medved D. (2011). Inhibition of cathepsin B activity attenuates extracellular matrix degradation and inflammatory breast cancer invasion. Breast Cancer Res..

[104] Mikhaylov G., Klimpel D., Schaschke N., Mikac U., Vizovisek M., Fonovic M., Turk V., Turk B., Vasiljeva O. (2014). Selective targeting of tumor and stromal cells by a nanocarrier system displaying lipidated cathepsin b inhibitor. Angew. Chem. Int. Ed..

[105] Alberto M., Sozzani S., Locati M., Allavena P., Sica A. (2002). Macrophage polarization: Tumor-associated macrophages as a paradigm for polarized M 2 mononuclear phagocytes. Trends Immunol..

[106] Weissleder R., Nahrendorf M., Pittet M.J. (2014). Imaging macrophages with nanoparticles. Nat. Mater..

[107] Ries C.H., Cannarile M.A., Hoves S., Benz J., Wartha K., Runza V., Rey-Giraud F., Pradel L.P., Feuerhake F., Klaman I. (2014). Targeting tumor-associated macrophages with anti-CSF-1R antibody reveals a strategy for cancer therapy. Cancer Cell.

[108] Masaki Y., Shimizu Y., Yoshioka T., Tanaka Y., Nishijima K., Zhao S., Higashino K., Sakamoto S., Numata Y., Yamaguchi Y. (2015). The accumulation mechanism of the hypoxia imaging probe “FMISO” by imaging mass spectrometry: Possible involvement of low-molecular metabolites. Sci. Rep..

[109] Lapi S.E., Lewis J.S., Dehdashti F. (2015). Evaluation of hypoxia with copper-labeled diacetyl-bis (*N*-methylthiosemicarbazone). Semin. Nucl. Med..

[110] Papadopoulou M.V., Pouremad R., Bloomer W.D., Wyrwicz A. (2006). Novel non-invasive probes for measuring tumor-hypoxia by 19F-magnetic resonance spectroscopy (19F-MRS). Studies in the SCCVII/C3H murine model. Anticancer Res..

[111] Cai Q., Yu T., Zhu W., Xu Y., Qian X. (2015). A turn-on fluorescent probe for tumor hypoxia imaging in living cells. Chem. Commun..

[112] Van Sluis R., Bhujwalla Z.M., Raghunand N., Ballesteros P., Alvarez J., Cerdán S., Galons J.P., Gillies R.J. (1999). In Vivo imaging of extracellular pH using 1H MRSI. Magn. Reson. Med..

[113] Moon B.F., Jones K.M., Chen L.Q., Liu P., Randtke E.A., Howison C.M., Pagel M.D. (2015). A comparison of iopromide and iopamidol, two acidoCEST MRI contrast media that measure tumor extracellular pH. Contrast Media Mol. Imaging.

[114] Kim D., Gao Z.G., Lee E.S., Bae Y.H. (2009). In Vivo evaluation of doxorubicin-loaded polymeric micelles targeting folate receptors and early endosomal pH in drug-resistant ovarian cancer. Mol. Pharm..

[115] Marelli U.K., Rechenmacher F., Sobahi T.R.A., Mas-Moruno C., Kessler H. (2013). Tumor targeting via integrin ligands. Front. Oncol..

[116] Golestani R., Zeebregts C.J., van Scheltinga A.G.T.T., Hooge M.N.L., van Dam G.M., Glaudemans A.W.J.M., Dierckx R.A.J.O., Tio R.A., Suurmeijer A.J.H., Boersma H.H. (2013). Feasibility of vascular endothelial growth factor imaging in human atherosclerotic plaque using 89Zr-bevacizumab positron emission tomography. Mol. Imaging.

[117] Chen K., Cai W., Li Z.-B., Wang H., Chen X. (2009). Quantitative PET imaging of VEGF receptor expression. Mol. Imaging Biol..

[118] Shi S., Yang K., Hong H., Chen F., Valdovinos H.F., Goel S., Barnhart T.E., Liu Z., Cai W. (2015). VEGFR targeting leads to significantly enhanced tumor uptake of nanographene oxide in vivo. Biomaterials.

[119] Kang C.M., Koo H.-J., Lee K.C., Choe Y.S., Choi J.Y., Lee K.-H., Kim B.-T. (2013). A vascular endothelial growth factor 121 (VEGF 121)-based dual PET/optical probe for in vivo imaging of VEGF receptor expression. Biomaterials.

[120] Santimaria M., Moscatelli G., Viale G.L., Giovannoni L., Neri G., Viti F., Leprini A., Borsi L., Castellani P., Zardi L. (2003). Immunoscintigraphic detection of the ED-B domain of fibronectin, a marker of angiogenesis, in patients with cancer. Clin. Cancer Res..

[121] Zhang F., Niu G., Lu G., Chen X. (2011). Preclinical lymphatic imaging. Mol. Imaging Biol..

[122] Lee S., Cha E.-J., Park K., Lee S.-Y., Hong J.-K., Sun I.-C., Kim S.Y., Choi K., Kwon I.C., Kim K. (2008). A Near-Infrared-Fluorescence-Quenched Gold-Nanoparticle Imaging Probe for In Vivo Drug Screening and Protease Activity Determination. Angew. Chem..

[123] Lee H., Lee K., Kim I.K., Park T.G. (2008). Synthesis, characterization, and in vivo diagnostic applications of hyaluronic acid immobilized gold nanoprobes. Biomaterials.

[124] Li Y., Foss C.A., Pomper M.G., Yu S.M. (2014). Imaging denatured collagen strands in vivo and ex vivo via photo-triggered hybridization of caged collagen mimetic peptides. J. Vis. Exp..

[125] Chopra A. (2012). Fibroblast Activation Protein α-Specific, Near-Infrared Peptide Probe (KGPGPNQC) Linked to Cy5. 5 and a Quencher Dye, QSY21.

[126] Wagstaff K., Jans D. (2006). Protein Transduction: Cell Penetrating Peptides and Their Therapeutic Applications. Curr. Med. Chem..

[127] Liu J.N., Bu W., Pan L.M., Zhang S., Chen F., Zhou L., Zhao K.L., Peng W., Shi J. (2012). Simultaneous nuclear imaging and intranuclear drug delivery by nuclear-targeted multifunctional upconversion nanoprobes. Biomaterials.

[128] Griset A.P., Walpole J., Liu R., Gaffey A., Colson Y.L., Grinstaff M.W. (2009). Expansile nanoparticles: Synthesis, characterization, and in vivo efficacy of an acid-responsive polymeric drug delivery system. J. Am. Chem. Soc..

[129] Zheng X.T., Ma X.Q., Li C.M. (2016). Highly efficient nuclear delivery of anti-cancer drugs using a bio-functionalized reduced graphene oxide. J. Colloid Interface Sci..

[130] Hao Y., Wang L., Zhang B., Li D., Meng D., Shi J., Zhang H., Zhang Z., Zhang Y. (2016). Manganese dioxide nanosheets-based redox/pH-responsive drug delivery system for cancer theranostic application. Int. J. Nanomed..

[131] Wang H., Liu G., Dong S., Xiong J., Du Z., Cheng X. (2015). A pH-responsive AIE nanoprobe as a drug delivery system for bioimaging and cancer therapy. J. Mater. Chem. B.

[132] Kumar B., Kulanthaivel S., Mondal A., Mishra S., Banerjee B., Bhaumik A., Banerjee I., Giri S. (2016). Mesoporous silica nanoparticle based enzyme responsive system for colon specific drug delivery through guar gum capping. Colloids Surf. B Biointerfaces.

[133] Yang Y., Aw J., Chen K., Liu F., Padmanabhan P., Hou Y., Cheng Z., Xing B. (2011). Enzyme-Responsive Multifunctional Magnetic Nanoparticles for Tumor Intracellular Drug Delivery and Imaging. Chem. Asian J..

[134] Zhang G., Gao J., Qian J., Zhang L., Zheng K., Zhong K., Cai D., Zhang X., Wu Z. (2015). Hydroxylated Mesoporous Nanosilica Coated by Polyethylenimine Coupled with Gadolinium and Folic Acid: A Tumor-Targeted T 1 Magnetic Resonance Contrast Agent and Drug Delivery System. ACS Appl. Mater. Interfaces.

[135] Louie A. (2010). Multimodality imaging probes: Design and challenges. Chem. Rev..

[136] Khan D.R., Webb M.N., Cadotte T.H., Gavette M.N. (2015). Use of targeted liposome-based chemotherapeutics to treat breast cancer. Breast Cancer Basic Clin. Res..

[137] Johnston M.J.W., Edwards K., Karlsson G., Cullis P.R. (2008). Influence of drug-to-lipid ratio on drug release properties and liposome integrity in liposomal doxorubicin formulations. J. Liposome Res..

[138] Ali Mohammadi Z., Foad Aghamiri S., Zarrabi A., Reza Talaie M. (2016). Liposomal Doxorubicin Delivery Systems: Effects of Formulation and Processing Parameters on Drug Loading and Release Behavior. Curr. Drug Deliv..

[139] Chang D.-K., Li P.-C., Lu R.-M., Jane W.-N., Wu H.-C. (2013). Peptide-mediated liposomal Doxorubicin enhances drug delivery efficiency and therapeutic efficacy in animal models. PLoS ONE.

[140] Liu Z., Xiong M., Gong J., Zhang Y., Bai N., Luo Y., Li L., Wei Y., Liu Y., Tan X. (2014). Legumain protease-activated TAT-liposome cargo for targeting tumours and their microenvironment. Nat. Commun..

[141] Awada A., Bondarenko I.N., Bonneterre J., Nowara E., Ferrero J.M., Bakshi A.V., Wilke C., Piccart M., Group C.S. (2014). A randomized controlled phase II trial of a novel composition of paclitaxel embedded into neutral and cationic lipids targeting tumor endothelial cells in advanced triple-negative breast cancer (TNBC). Ann. Oncol..

[142] Muhamad I.I., Selvakumaran S. (2014). Designing Polymeric Nanoparticles for Targeted Drug Delivery System Outline. Nanomedicine.

[143] Luk B.T., Zhang L. (2014). Current advances in polymer-based nanotheranostics for cancer treatment and diagnosis. ACS Appl. Mater. Interfaces.

[144] Napp J., Behnke T., Fischer L., Würth C., Wottawa M., Katschinski D.M., Alves F., Resch-Genger U., Schäferling M. (2011). Targeted luminescent near-infrared polymer-nanoprobes for in vivo imaging of tumor hypoxia. Anal. Chem..

[145] Jain T.K., Richey J., Strand M., Leslie-Pelecky D.L., Flask C.A., Labhasetwar V. (2008). Magnetic nanoparticles with dual functional properties: Drug delivery and magnetic resonance imaging. Biomaterials.

[146] Gao H., Wang Y.N., Fan Y.G., Ma J.B. (2005). Synthesis of a biodegradable tadpole-shaped polymer via the coupling reaction of polylactide onto mono(6-(2-aminoethyl)amino-6-deoxy)-β-cyclodextrin and its properties as the new carrier of protein delivery system. J. Control. Release.

[147] Fernandez-Fernandez A., Manchanda R., McGoron A.J. (2011). Theranostic Applications of Nanomaterials in Cancer: Drug Delivery, Image-Guided Therapy, and Multifunctional Platforms. Appl. Biochem. Biotechnol..

[148] Gobbo O.L., Sjaastad K., Radomski M.W., Volkov Y., Prina-Mello A. (2015). Magnetic Nanoparticles in Cancer Theranostics. Theranostics.

[149] Shi J., Kantoff P.W., Wooster R., Farokhzad O.C. (2016). Cancer nanomedicine: Progress, challenges and opportunities. Nat. Rev. Gancer.

[150] Meyers J.D., Doane T., Burda C., Basilion J.P. (2013). Nanoparticles for imaging and treating brain cancer. Nanomedicine.

[151] Lu J., Liong M., Li Z., Zink J.I., Tamanoi F. (2010). Biocompatibility, Biodistribution, and Drug-Delivery Efficiency of Mesoporous Silica Nanoparticles for Cancer Therapy in Animals. Small.

[152] Xiao Q., Zheng X., Bu W., Ge W., Zhang S., Chen F., Xing H., Ren Q., Fan W., Zhao K. (2013). A Core/Satellite Multifunctional Nanotheranostic for in vivo Imaging and Tumor Eradication by Radiation/Photothermal Synergistic Therapy. J. Am. Chem. Soc..

[153] Hu Q., Katti P.S., Gu Z. (2014). Enzyme-responsive nanomaterials for controlled drug delivery. Nanoscale.

